# M^6^A‐mediated upregulation of lncRNA TUG1 in liver cancer cells regulates the antitumor response of CD8^+^ T cells and phagocytosis of macrophages

**DOI:** 10.1002/advs.202400695

**Published:** 2024-07-09

**Authors:** Qing Xi, Guangze Yang, Xue He, Hao Zhuang, Li Li, Bing Lin, Lingling Wang, Xianyang Wang, Chunqiang Fang, Qiurui Chen, Yongjie Yang, Zhaoan Yu, Hao Zhang, Wenqian Cai, Yan Li, Han Shen, Li Liu, Rongxin Zhang

**Affiliations:** ^1^ Department of Gastroenterology and Hepatology The First Affiliated Hospital of Guangdong Pharmaceutical University Guangzhou 510080 China; ^2^ School of Biomedical Sciences and Engineering South China University of Technology Guangzhou 511442 China; ^3^ Laboratory of Immunology and Inflammation Department of Immunology Key Laboratory of Immune Microenvironment and Diseases of Educational Ministry of China Tianjin Medical University Tianjin 300070 China; ^4^ Laboratory of Immunology and Inflammation Department of Biotechnology School of Life Sciences and Biopharmaceutics Guangdong Provincial Key Laboratory of Advanced Drug Delivery Guangdong Provincial Engineering Center of Topical Precise Drug Delivery System Guangdong Pharmaceutical University Guangzhou 51006 China; ^5^ Department of Hepatobiliopancreatic Surgery The Affiliated Cancer Hospital of Zhengzhou University & Henan Cancer Hospital Zhengzhou 450008 China; ^6^ Department of Bioscience School of Life Sciences and Biopharmaceutics Guangdong Pharmaceutical University Guangzhou 51006 China; ^7^ Department of Radiology The University of Texas Southwestern Medical Center 5323 Harry Hines Blvd. Dallas TX 75390 USA

**Keywords:** antitumor response, CD47, Hepatocellular carcinoma, LncRNA TUG1, PD‐L1

## Abstract

Tumor immune evasion relies on the crosstalk between tumor cells and adaptive/innate immune cells. Immune checkpoints play critical roles in the crosstalk, and immune checkpoint inhibitors have achieved promising clinical effects. The long non‐coding RNA taurine‐upregulated gene 1 (TUG1) is upregulated in hepatocellular carcinoma (HCC). However, how TUG1 is upregulated and the effects on tumor immune evasion are incompletely understood. Here, METTL3‐mediated m^6^A modification led to TUG1 upregulation is demonstrated. Knockdown of TUG1 inhibited tumor growth and metastasis, increased the infiltration of CD8^+^ T cells and M1‐like macrophages in tumors, promoted the activation of CD8^+^ T cells through PD‐L1, and improved the phagocytosis of macrophages through CD47. Mechanistically, TUG1 regulated PD‐L1 and CD47 expressions by acting as a sponge of miR‐141 and miR‐340, respectively. Meanwhile, TUG1 interacted with YBX1 to facilitate the upregulation of PD‐L1 and CD47 transcriptionally, which ultimately regulated tumor immune evasion. Clinically, TUG1 positively correlated with PD‐L1 and CD47 in HCC tissues. Moreover, the combination of Tug1‐siRNA therapy with a Pdl1 antibody effectively suppressed tumor growth. Therefore, the mechanism of TUG1 in regulating tumor immune evasion is revealed and can inform existing strategies targeting TUG1 for enhancing HCC immune therapy and drug development.

## Graphical abstract

METTL3 mediates m^6^A modification leading to upregulation of lncRNA TUG1 in HCC. Knockdown of TUG1 promotes anti‐tumor immunity by restoring the activation of CD8^+^ T cells and improves phagocytosis of tumor cells by macrophages. TUG1 sponges miR‐141 and miR‐340, and interacts with YBX1 to regulate PD‐L1 and CD47, which provides a promising immunotherapeutic strategy for the treatment of HCC.

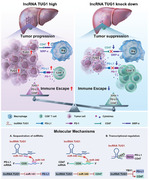

## 1 Introduction

Hepatocellular carcinoma (HCC) is the most common form of primary liver cancer, which is a global health challenge with growing incidence and mortality.^[^ ^1^ ^]^ Several factors are related to the progression of HCC, such as chronic hepatitis B and hepatitis C (accounting for 80%), alcohol addiction, dietary toxins, and metabolic liver disease.^[^ ^2^ ^]^ Although surgical treatments have long been the mainstay treatments for HCC, 70% of patients develop recurrences after resection.^[^ ^3^ ^]^ In addition, most patients are not diagnosed until an advanced stage, facing limited treatment options and poor prognosis.^[^ ^4^ ^]^ Therefore, it is urgent to develop effective medical therapies for HCC.

Over the past few decades, immune checkpoint blockades (ICBs) have emerged as an effective treatment option for a subset of HCC patients. However, the majority of patients do not respond, and the response rate is only ≈20%.^[^ ^5^ ^]^ There is a complex ecosystem involving the tumor immune microenvironment of HCC, and the crosstalk between tumor cells and different populations of adaptive and innate immune cells influences tumor immune escape and immunotherapy response. The CD8^+^ T cells of the adaptive immune system are the main antitumor effector cells.^[^ ^6^ ^]^ They must confront various barriers including intrinsic checkpoints, such as programmed cell death 1/ programmed cell death‐ligand 1 (PD1/PD‐L1),CD28 molecule (CD28) and cytotoxic T‐lymphocyte‐associated protein 4 (CTLA‐4). PD‐L1 also known as CD274 or B7‐H1, is one of the well‐known immune checkpoints, which presents a critical “don't find me” signal in adaptive immune response: it can bind to PD‐1 to inhibit T cells activation, consequently inducing tumor immune escape.^[^ ^7^ ^]^ In addition, innate immune cells also play critical roles in the activation of antitumor immunity. Tumor‐associated macrophages (TAMs) represent one type of innate immune cells that extensively infiltrate the tumor immune microenvironment and are associated with poor prognosis.^[^ ^8^ ^]^ Tumor cells express anti‐phagocytic signals such as cluster of differentiation 47 (CD47) to escape phagocytosis by macrophages. CD47, also known as IAP, MER6, or OA3, is a pivotal signal of the innate immune response and critical in the regulation of adaptive immune response.^[^ ^9^ ^]^ It plays a key role in delivering “don't eat me” signals to macrophages by binding to signal‐regulatory protein alpha (SIRPα), which leads to the inhibition of phagocytosis.^[^ ^10^ ^]^ Both PD‐L1 and CD47 are frequently overexpressed on HCC cells.^[^ ^11^, ^12^ ^]^ Recently, emerging evidence suggests that down‐regulation of PD‐L1 and CD47 in tumor cells has significant immunotherapeutic effects.^[^ ^13^, ^14^ ^]^ To further elucidate the mechanisms of tumor immune escape and provide new strategies for existing immunotherapy, an in‐depth study is urgently needed.

Accumulating evidence suggests that long non‐coding RNAs (lncRNAs) are closely related to tumor immunity.^[^ ^15^ ^]^ A better understanding of the mechanisms underlying how lncRNAs regulate key immune checkpoints will provide new ideas for immunotherapy. The long non‐coding RNA TUG1 was first identified as being required for the differentiation of mouse retinal cells,^[^ ^16^ ^]^ it is also involved in the regulation of mitochondrial bioenergetics in diabetic nephropathy,^[^ ^17^ ^]^ as well as male fertility.^[^ ^18^ ^]^ TUG1 is upregulated in a variety of cancers including HCC, and the cancer biological functions of TUG1 have been well studied.^[^ ^19^, ^20^ ^]^ However, how TUG1 is upregulated in cancers and the effects of its upregulation on the tumor immune microenvironments are incompletely understood.

In this study, we demonstrated that lncRNA TUG1 upregulation was mediated by METTL3‐mediated m^6^A modification and examined the underlying mechanism of how TUG1 in liver cancer cells regulates the antitumor response of CD8^+^ T cells and phagocytosis of macrophages. Inhibition of TUG1 could become a promising strategy to restore antitumor immunity by regulating PD‐L1 and CD47, which could provide new ideas for designing effective immunotherapeutic strategies and improve the therapeutic effect of current immune checkpoint inhibitors for HCC.

## 2 Results

### 2.1 M^6^A‐Mediated Upregulation of TUG1 is Related to Worse Prognosis in HCC

**Figure 1:**
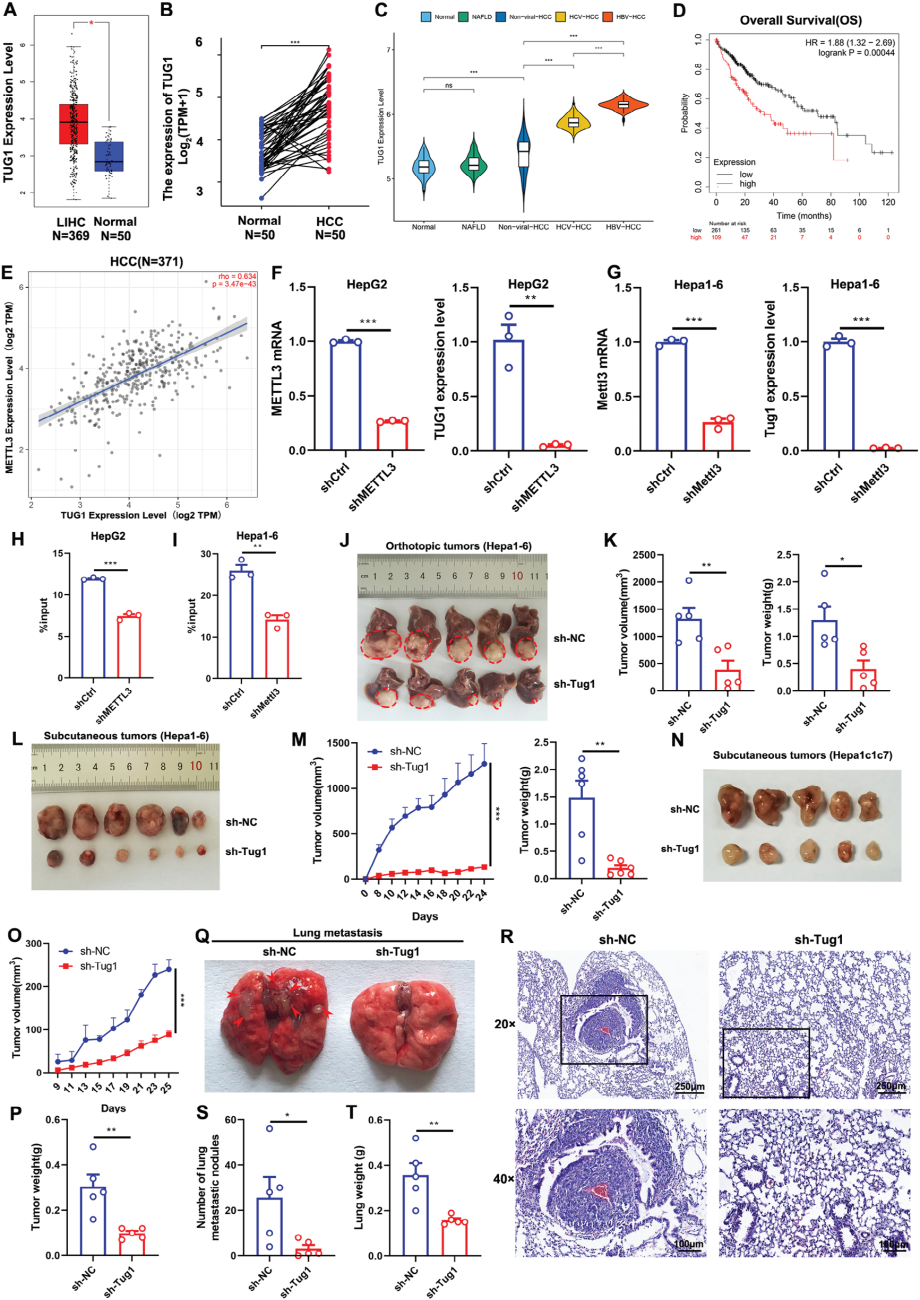
M^6^A‐mediated upregulation of TUG1 is related to worse prognosis, tumor growth, and metastasis in HCC. Comparison of TUG1 expression in unpaired A) and paired B) HCC tissues with normal tissues. C) The expression of TUG1 among 112 HBV‐HCCs, 91 HCV‐HCCs, 112 non‐viral‐HCCs, 76 NAFLD patients and 91 healthy controls. D) Overall survival analysis of TUG1 in HCC patients (*n* = 370). E) The correlation of TUG1 and METTL3 in HCC patients was measured by Spearman's correlation analysis (*n* = 371). F) The expressions of METTL3 and TUG1 in the control and sh‐METTL3 HepG2 cells (*n* = 3). G) The expressions of Mettl3 and Tug1 in the control and sh‐Mettl3 Hepa1‐6 cells (*n* = 3). H) MeRIP‐qPCR shows that the m^6^A modification of TUG1 decreases along with METTL3 downregulation in the control and sh‐METTL3 HepG2 cells (*n* = 3). I) MeRIP‐qPCR shows that the m^6^A modification of Tug1 decreases along with Mettl3 downregulation in the control and sh‐Mettl3 Hepa1‐6 cells (*n* = 3). J) Representative images of tumors from the Hepa1‐6 orthotopic HCC mouse models are outlined by red circles. K) Final tumor volumes and tumor weights (*n* = 5). L) Representative images of tumors from the Hepa1‐6 subcutaneous HCC mouse models. M) Tumor growth curves and final tumor weights (*n* = 6). N) Representative images of tumors from the Hepa1c1c7 subcutaneous HCC mouse models. O) Tumor growth curves (*n* = 5). P) Final tumor weights (*n* = 5). Q) Representative images of lungs metastasis, with the metastatic nodules are indicated by red arrows. R) Representative H&E‐stained images of the lung sections. S) The quantification of lung metastatic nodules (*n* = 5). T) The weight of lungs (*n* = 5). Data are presented as the mean ± SEM. Data were statistically analyzed using unpaired two‐tailed Student's t‐test (A, B, C, F, G, H, I, K, M, P, S and T) or two‐way ANOVA (M and O) or Kaplan‐Meier survival analysis (D) or Spearman's correlation analysis (E). *, *p* < 0.05; **, *p* < 0.01; ***, *p* < 0.001.

To explore the potential involvement of TUG1 in HCC, we compared TUG1 expression in both unpaired and paired HCC tissues to normal tissues in the TCGA, TUG1 was overexpressed in HCC tissues (**Figure** **1**A,B). In addition, among 112 HBV‐HCCs, 91 HCV‐HCCs, 112 non‐viral‐HCCs, 76 NAFLD patients, and 91 healthy controls, TUG1 expression was the highest in HBV‐HCCs (Figure 1C). Furthermore, the high expression of TUG1 indicated poorer overall survival in HCC patients (Figure 1D). As m^6^A modification in lncRNAs plays a critical role in tumor epigenetic regulation, we further analyzed the relevance of TUG1 and the m^6^A methyltransferase METTL3. The results showed that METTL3 positively correlated with TUG1 in HCC (Figure 1E). In addition, the silence of METTL3 decreased TUG1 expression (Figure 1F) and reduced the m^6^A modification of TUG1 in HepG2 cells (Figure 1H), the silence of Mettl3 decreased Tug1 expression (Figure 1G) and reduced the m^6^A modification of Tug1 in Hepa1‐6 cells (Figure 1I). Taken together, the results showed that METTL3 plays a critical role in maintaining a high level of TUG1. In patients suffering from HCC, the high expression of TUG1 indicated poorer overall survival. An in‐deep study of the mechanism of TUG1 may provide new options for existing therapeutic strategies.

### 2.2 Knockdown of Tug1 Inhibits Tumor Growth and Metastasis

To evaluate the role of Tug1 in the tumor growth of HCC, we performed an orthotopic mouse model of HCC with sh‐NC and sh‐Tug1 Hepa1‐6 cells. The tumor volume and tumor weight decreased significantly in sh‐Tug1 Hepa1‐6 cell‐implanted mice (Figure 1J,K). Similarly, the downregulation of Tug1 reduced both the tumor volume and tumor weight significantly in subcutaneous mouse models (Figure 1L,M). We also performed a subcutaneous mouse model of HCC with sh‐NC and sh‐Tug1 Hepa1c1c7 cells, and the tumor volume and tumor weight decreased significantly in sh‐Tug1 Hepa1c1c7 cell‐implanted mice (Figure 1N–P). Furthermore, to evaluate the role of Tug1 in the tumor metastasis of HCC, the lungs of the experimental mice were acquired and lung metastatic nodules were stained with hematoxylin‐eosin (H&E) staining. The results showed that the lung metastatic nodules were much less (Figure 1Q–S), and the lung weight was much lighter in the Tug1 knocked‐down group (Figure 1T). Next, we explored the biological function of Tug1 in vitro. The results showed that the downregulation of Tug1 (Figure S1A, Supporting Information) inhibited cancer cell proliferation, migration, and invasion (Figure S1B–F, Supporting Information), and promoted cell apoptosis (Figure S1G,H, Supporting Information).

Altogether, m^6^A‐mediated upregulation of Tug1 is related to a worse prognosis in HCC, and the knockdown of Tug1 inhibits tumor growth and metastasis of HCC. However, the role of Tug1 upregulation in the tumor immune microenvironments is incompletely understood. Further study on the mechanism of Tug1 in regulating tumor immunity may bring new hope for patients who suffer from HCC.

### 2.3 Knockdown of Tug1 Notably Promotes Antitumor Immunity

**Figure 2:**
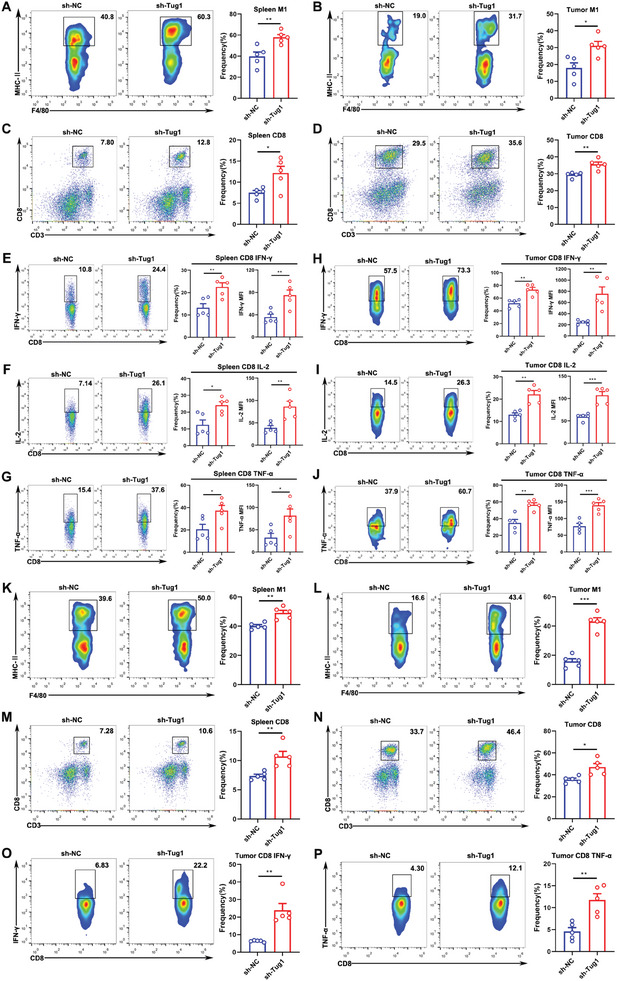
Knockdown of Tug1 notably promotes antitumor immunity. The frequency of M1‐like (CD11b^+^F4/80^+^MHC‐II^+^) macrophages in the spleens A) and tumors B) of mice bearing sh‐NC and sh‐Tug1 Hepa1‐6 cells (*n* = 5). The frequencies of CD8^+^ T cells in the spleens C) and tumors D) of mice bearing sh‐NC and sh‐Tug1 Hepa1‐6 cells (*n* = 5). The frequencies and MFI of cytokines (IFN‐γ, IL‐2, and TNF‐α) secreted by CD8^+^ T cells in the spleens E–G) and tumors H–J) of mice bearing sh‐NC and sh‐Tug1 Hepa1‐6 cells (*n* = 5). The frequencies of M1‐like (CD11b^+^F4/80^+^MHC‐II^+^) macrophages in the spleens K) and tumors L) of mice bearing sh‐NC and sh‐Tug1 Hepa1c1c7 cells (*n* = 5). The frequencies of CD8^+^ T cells in the spleens M) and tumors N) of mice bearing sh‐NC and sh‐Tug1 Hepa1c1c7 cells (*n* = 5). O, P) The frequencies of IFN‐γ and TNF‐α secreted by CD8^+^ T cells in the tumors of mice bearing sh‐NC and sh‐Tug1 Hepa1c1c7 cells (*n* = 5). Results are represented as the mean ± SEM. Statistical analysis was performed using the unpaired two‐tailed Student's t‐test. *, *p* < 0.05; **, *p* < 0.01; ***, *p* < 0.001.

To explore whether the knockdown of Tug1 could promote antitumor immunity in HCC, we established an orthotopic mouse model of HCC with sh‐NC and sh‐Tug1 Hepa1‐6 cells and performed flow cytometry. M1‐like macrophages (CD11b^+^F4/80^+^MHC‐II^+^) clearly increased in the spleens and tumors of sh‐Tug1 Hepa1‐6 cell‐bearing mice (**Figure** **2**A,B). Consistently, CD8^+^ T lymphocytes were also clearly increased (Figure 2C,D) and showed stronger activation phenotypes with more production of IFN‐γ, IL‐2, and TNF‐α in both the spleens and tumors of sh‐Tug1 Hepa1‐6 cell‐bearing mice (Figure 2E–J). In addition, CD4^+^ T cells clearly increased (Figure S2A,B, Supporting Information), with increased secretion of IFN‐γ and IL‐2 in both the spleens and tumors of sh‐Tug1 Hepa1‐6 cell‐bearing mice (Figure S2C–F, Supporting Information). Furthermore, we established an orthotopic mouse model of HCC with sh‐NC and sh‐Tug1 Hepa1c1c7 cells, and performed flow cytometry. Similarly, M1‐like macrophages clearly increased in the spleens and tumors of sh‐Tug1 Hepa1c1c7 cell‐bearing mice (Figure 2K,L). Consistently, CD8^+^ T lymphocytes were clearly increased in both the spleens and tumors of sh‐Tug1 Hepa1c1c7 cell‐bearing mice (Figure 2M,N), and CD8^+^ T cells in the tumors of sh‐Tug1 Hepa1c1c7 cell‐bearing mice showed stronger activation phenotypes with more production of IFN‐γ and TNF‐α (Figure 2O,P). Therefore, m^6^A‐mediated upregulation of Tug1 is closely related to tumor immunity and may serve as a novel immunotherapy strategy for clinical patients suffering from HCC.

### 2.4 Tug1 in Tumor Cells Regulates the Antitumor Immune Response of CD8^+^ T Cells and Macrophages

**Figure 3:**
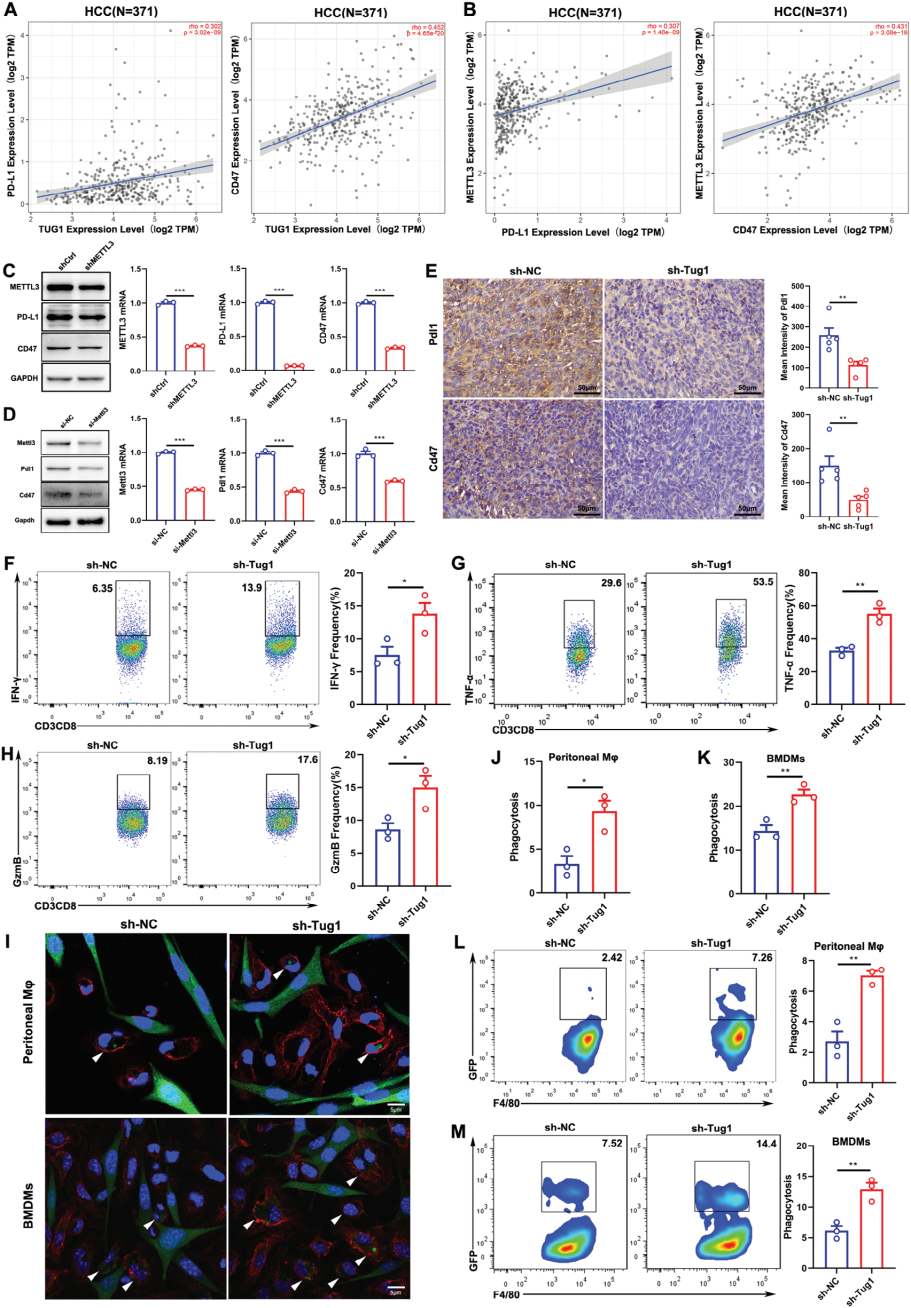
Tug1 in tumor cells regulates the antitumor immune response of CD8^+^ T cells and phagocytosis of macrophages through PD‐L1 and CD47, respectively. A) The correlation of TUG1 with PD‐L1 and CD47 in HCC patients (*n* = 371). B) The correlation of METTL3 with PD‐L1 and CD47 in HCC patients (*n* = 371). C) The expressions of PD‐L1 and CD47 at the protein and mRNA levels in the control and sh‐METTL3 HepG2 cells. D) The expressions of Pdl1 and Cd47 at the protein and mRNA levels in the control and sh‐Mettl3 Hepa1‐6 cells. E) Representative images of IHC staining and mean intensities of PD‐L1 and CD47 (*n* = 5. Magnification: 40 ×). F–H) The frequencies of IFN‐γ, TNF‐α, and GzmB in CD8^+^ T cells co‐cultured with sh‐NC or sh‐Tug1 Hepa1‐6 cells (*n* = 3). I) Representative images from immunofluorescence (IF) staining of peritoneal cavity‐derived macrophages and BMDMs engulfing cancer cells. The white arrows indicate macrophages that phagocytose cancer cells. Macrophages are shown in red (F4/80^+^), cancer cells are shown in green (GFP^+^) and nuclei are shown in blue (DAPI). Magnification: 100 ×. J, K) Statistical analysis of phagocytosis by macrophages as detected via IF staining (*n* = 3). L, M) Representative plots and statistical analysis of phagocytosis by macrophages derived from the peritoneal cavity and bone marrow as detected using a flow cytometer (*n* = 3). Results are represented as the mean ± SEM. Data were statistically analyzed using unpaired two‐tailed Student's t‐test (C‐H, J‐M) or Spearman's correlation analysis (A‐B).*, *p* < 0.05; **, *p* < 0.01; ***, *p* < 0.001.

To explore the cellular and molecular mechanisms underlying how TUG1 regulates antitumor immunity, the correlation of TUG1 with critical immune checkpoints was analyzed. We discovered that TUG1 positively correlated with both PD‐L1 and CD47 in HCC (**Figure** **3**A). Additionally, METTL3 positively correlated with both PD‐L1 and CD47 in HCC (Figure 3B), and the downregulation of METTL3 significantly decreased PD‐L1 and CD47 expressions in HepG2 cells (Figure 3C). We also confirmed the results with another 2 siRNAs, which showed that the downregulation of METTL3 significantly decreased PD‐L1 and CD47 expressions at both the protein and mRNA levels in HepG2 cells (Figure S3A–D, Supporting Information). The results in Hepa1‐6 cells also showed that the downregulation of Mettl3 significantly decreased Pdl1 and Cd47 expressions at both the protein and mRNA levels (Figure 3D). Similarly, the results in LM3 cells showed that the downregulation of METTL3 significantly decreased PD‐L1 and CD47 expressions at both the protein and mRNA levels (Figure S3E,F, Supporting Information). Furthermore, the immunohistochemistry (IHC) results indicated that both Pdl1 and Cd47 expressions decreased in the tumors obtained from sh‐Tug1 Hepa1‐6 cell‐bearing mice (Figure 3E). Previous research has shown that PD‐L1 interacts with PD‐1 to inhibit the activation of T cells. Therefore, we further co‐cultured isolated CD8^+^ T cells with sh‐NC or sh‐Tug1 Hepa1‐6 cells, and the secretion of IFN‐γ, TNF‐α, and GzmB were increased in CD8^+^ T cells co‐cultured with sh‐Tug1 Hepa1‐6 cells (Figure 3F–H). We also co‐cultured isolated CD8^+^ T cells with sh‐NC or sh‐Tug1 Hepa1c1c7 cells, and similarly, the secretions of IFN‐γ, TNF‐α and IL‐2 were increased in CD8^+^ T cells co‐cultured with sh‐Tug1 Hepa1c1c7 cells (Figure S4A–C, Supporting Information), which indicated that the knockdown of Tug1 could restore the activation of CD8^+^ T cells. In addition, CD47 is also crucial for cancer cells to evade immune clearance, and abolishing the binding of CD47 to SIRPα could induce target cell phagocytosis by macrophages. We further co‐cultured peritoneal cavity‐derived or bone marrow‐derived macrophages (BMDMs) with sh‐NC or sh‐Tug1 Hepa1‐6 cells. Representative immunofluorescence images showed that the downregulation of Tug1 significantly increased the phagocytosis of cancer cells by macrophages derived from both the peritoneal cavity and bone marrow (Figure 3I–K). The phagocytosis of sh‐Tug1 Hepa1‐6 cells by peritoneal cavity‐derived macrophages (Figure 3L) and BMDMs (Figure 3M) increased significantly, as detected via flow cytometry. We also co‐cultured peritoneal cavity‐derived macrophages with sh‐NC or sh‐Tug1 Hepa1c1c7 cells, and the phagocytosis of sh‐Tug1 Hepa1c1c7 cells by peritoneal cavity‐derived macrophages increased significantly (Figure S4D, Supporting Information).

Taken together, m^6^A‐mediated upregulation of Tug1 is closely related to tumor immunity, and it could inhibit the antitumor immune response of CD8^+^ T cells by promoting Pdl1 expression, and inhibiting the phagocytosis function of macrophages toward cancer cells by promoting Cd47 expression. Further studying the specific molecular mechanism involved in this process will bring new hope to patients suffering from HCC.

### 2.5 Tug1 Acts as a microRNA Sponge to Promote Pdl1 and Cd47 Expressions, Thereby Regulating the Antitumor Immune Response of CD8^+^ T Cells and Phagocytosis of Macrophages

**Figure 4:**
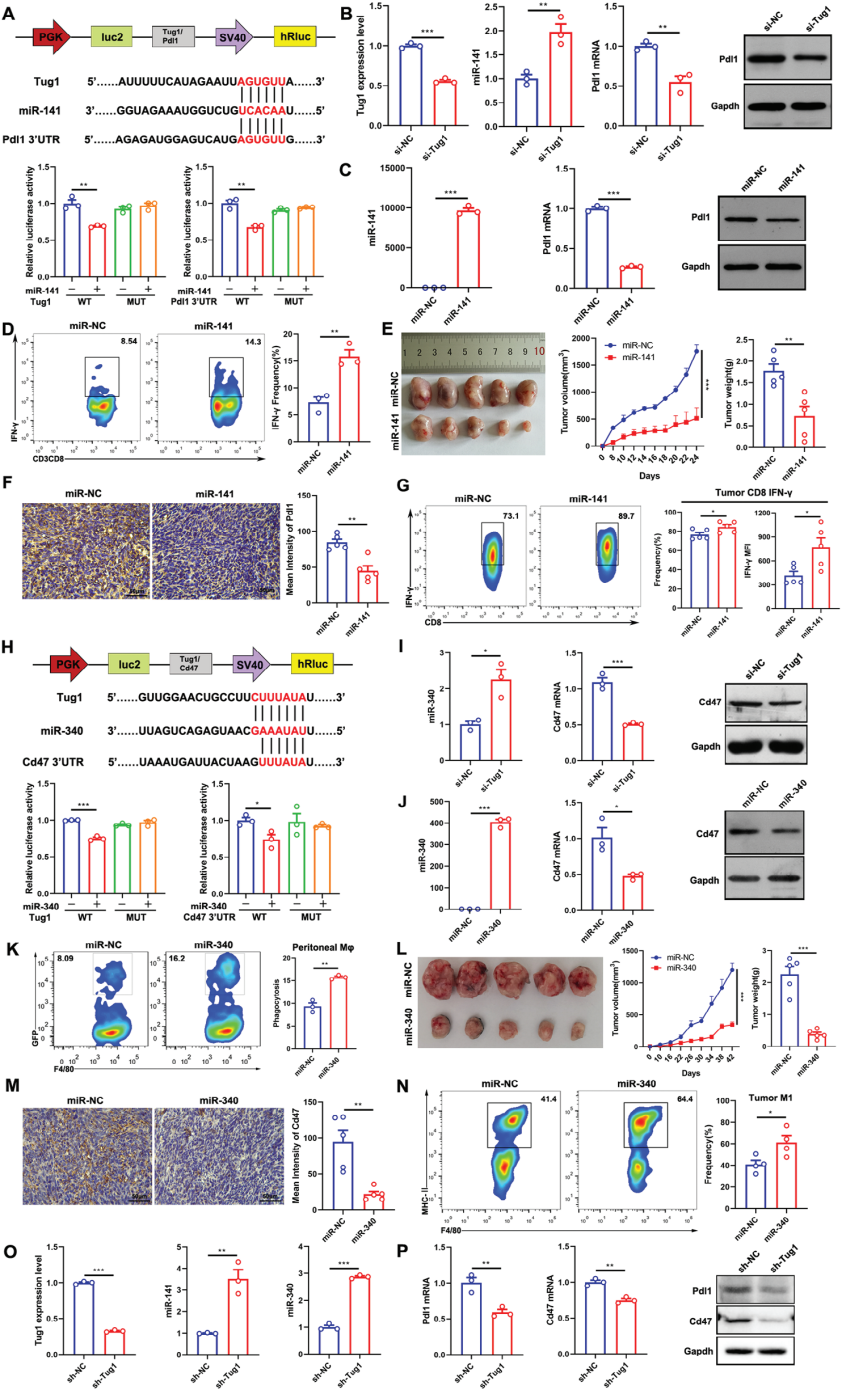
Tug1 acts as a microRNA sponge to promote PD‐L1 and CD47 expressions, thereby regulating the antitumor immune response of CD8^+^ T cells and phagocytosis of macrophages. A) The luciferase reporter assays verify the interaction sites of miR‐141 with Tug1 and Pdl1 (*n* = 3). B) The expressions of Tug1, miR‐141, and Pdl1 in the control and si‐Tug1‐transfected Hepa1‐6 cells. C) The expression of miR‐141 and Pdl1 in the control and miR‐141 mimic‐transfected Hepa1‐6 cells. D) The production frequency of IFN‐γ in CD8^+^ T cells co‐cultured with miR‐NC or miR‐141‐overexpressed Hepa1‐6 cells (*n* = 3). E) Representative images of tumors, tumor growth curves, and tumor weights from mice bearing miR‐NC and miR‐141‐overexpressed Hepa1‐6 cells bearing mice (*n* = 5). F) Immunohistochemistry (IHC) staining and mean intensity of Pdl1 (*n* = 5. Magnification: 40 ×). G) The secretion of IFN‐γ in CD8^+^ T cells from the tumors of mice bearing miR‐NC and miR‐141‐overexpressed Hepa1‐6 cells (*n* = 5). H) The luciferase reporter assays verified the interaction sites of miR‐340 with Tug1 and Cd47 (*n* = 3). I) The expressions of miR‐340 and Cd47 in the control and si‐Tug1‐transfected Hepa1‐6 cells. J) The expressions of miR‐340 and Cd47 in the control and miR‐340 mimic‐transfected Hepa1‐6 cells. K) Representative plots of flow cytometry and statistical analysis of F4/80^+^ macrophages phagocytosing miR‐NC or miR‐340‐overexpressed Hepa1‐6 cells (*n* = 3). L) Representative images of tumors, tumor growth curves, and tumor weights from mice bearing miR‐NC and miR‐340‐overexpressed Hepa1‐6 cells (*n* = 5). M) Immunohistochemistry (IHC) staining of Cd47 (*n* = 5. Magnification: 40 ×). N) The frequency of M1‐like macrophages in the tumors obtained from mice bearing miR‐NC and miR‐340‐overexpressed Hepa1‐6 cells (*n* = 4). O) The expression of miR‐340 and miR‐141 were upregulated in sh‐Tug1 Hepa1c1c7 cells. P) Pdl1 and Cd47 expression at both mRNAs and protein levels in sh‐Tug1 Hepa1c1c7 cells. Results are represented as the mean ± SEM. Data were statistically analyzed using unpaired two‐tailed Student's t‐test or two‐way ANOVA (E, L). *, *p* < 0.05; **, *p* < 0.01; ***, *p* < 0.001.

Accumulating evidence shows that lncRNAs could act as microRNA sponges to exert a “sponge‐like” function, and consequently regulate gene expression.^[^ ^21^ ^]^ We used the online bio information database ENCORI/starBase to predict the potential binding sites of miRNAs in the 3′UTR of Pdl1. Then, we used DIANA‐LncBase to predict the binding of miR‐141 to Tug1. We analyzed the sequences and found that miR‐141 has potential binding sites in both Tug1 and the 3′UTR of Pdl1; the luciferase activity of wt‐Tug1 was effectively inhibited by miR‐141, but the luciferase activity of mut‐Tug1 did not change, while the overexpression of miR‐141 inhibited the luciferase reporter activity of the wild‐type but not mutant Pdl1 3′UTR, suggesting that miR‐141 could target to both Tug1 and the 3′UTR of Pdl1 directly (**Figure** **4**A). We further found that miR‐141 was upregulated in Tug1 knocked‐down Hepa1‐6 cells, while the knockdown of Tug1 decreased Pdl1 expression (Figure 4B). Additionally, we found that overexpression of miR‐141 decreased the expression of Pdl1 (Figure 4C). These findings indicate that Tug1 sponges miR‐141 and promotes the expression of the miR‐141 target gene, Pdl1. As knockdown of Tug1 could restore the activation of CD8^+^ T cells, we attempted to evaluate the impact of overexpression of miR‐141 in Hepa1‐6 cells on the activation of CD8^+^ T cells. We found that the secretion of IFN‐γ increased in CD8^+^ T cells co‐cultured with miR‐141‐ overexpressing cells (Figure 4D). As Tug1 sponges miR‐141 and promotes Pdl1 in vitro, we further explored whether miR‐141 was involved in the regulation of tumor growth in vivo. The results showed that miR‐141 inhibited tumor growth and Pdl1 expression significantly (Figure 4E,F). The CD8^+^ T cells from the tumors of Hepa1‐6 cell‐bearing mice overexpressing miR‐141 showed stronger activation phenotypes with more production of IFN‐γ (Figure 4G), IL‐2 and TNF‐α (Figure S5A,B, Supporting Information). CD8^+^ T cells from the spleens of Hepa1‐6 cell‐bearing mice overexpressing miR‐141 also showed stronger activation phenotypes with more production of IFN‐γ, IL‐2, and TNF‐α (Figure S5C–E, Supporting Information). Altogether, Tug1 could promote Pdl1 expression by sponging miR‐141, consequently inhibiting the function of CD8^+^ T cells, thus ultimately playing a critical role in regulating antitumor immune response in HCC.

As Tug1 inhibits the phagocytosis function of macrophages toward cancer cells by promoting Cd47 expression, we further used the ENCORI/starBase to predict the potential binding sites of miRNAs in the 3′UTR of Cd47. Then, we used DIANA‐LncBase to predict the binding of miR‐340 to Tug1. The results showed that miR‐340 has binding sites in both Tug1 and the 3′UTR of Cd47. The luciferase activity of wt‐Tug1 was effectively repressed by miR‐340, while that of mut‐Tug1 did not change, and an overexpression of miR‐340 inhibited the luciferase reporter activity of the wild type but not mutant Cd47 3'UTR, which suggested that miR‐340 could target both Tug1 and Cd47 3′UTR directly (Figure 4H). We further found that miR‐340 was upregulated in Tug1 knocked‐down Hepa1‐6 cells, while the downregulation of Tug1 decreased Cd47 expression (Figure 4I). Additionally, we found that miR‐340 decreased Cd47 expression (Figure 4J). These findings indicate that Tug1 promotes Cd47 expression by acting as a sponge of miR‐340 via direct interactions. As the knockdown of Tug1 promoted the phagocytosis function of macrophages toward cancer cells, we attempted to evaluate the impact of overexpression of miR‐340 in Hepa1‐6 cells on the phagocytosis of macrophages. The results demonstrated that the phagocytosis of miR‐340‐overexpressing cells by peritoneal cavity‐derived macrophages increased significantly (Figure 4K). The phagocytosis of miR‐340‐overexpressing cells by BMDMs also increased (Figure S6A, Supporting Information). As Tug1 sponges miR‐340 and promotes Cd47 in vitro, we further explored whether miR‐340 plays a critical role in regulating tumor growth in vivo. We found that miR‐340 inhibited tumor growth and Cd47 expression significantly (Figure 4L,M). M1‐like macrophages from the tumors of Hepa1‐6 cell‐bearing mice overexpressing miR‐340 clearly increased (Figure 4N). We also found that M1‐like macrophages from the spleens of Hepa1‐6 cell‐bearing mice overexpressing miR‐340 clearly increased (Figure S6B, Supporting Information). Taken together, Tug1 could promote Cd47 expression by sponging miR‐340, and consequently inhibit the phagocytosis function of macrophages toward cancer cells, thus playing a critical role in the regulation of antitumor immune response.

To further confirm the mechanism of Tug1 in regulating antitumor immunity, we used another 2 siRNAs to confirm the regulation of Pdl1 and Cd47 by Tug1, the results showed that the downregulation of Tug1 significantly decreased Pdl1 and Cd47 expressions at both the protein and mRNA levels (Figure S7A–D, Supporting Information). We also explored the regulation with Hepa1c1c7 cells and found that both miR‐340 and miR‐141 were upregulated in Tug1‐downregulated Hepa1c1c7 cells (Figure 4O), while the expressions of Pdl1 and Cd47 decreased (Figure 4P). Overall, Tug1 sponges miR‐141 to promote the expression of Pdl1 and regulate the antitumor immune response of CD8^+^ T cells, and act as miR‐340 sponges to promote the expression of Cd47 and regulate phagocytosis of macrophages, thus ultimately playing a critical role in regulating the antitumor immune response in HCC.

### 2.6 TUG1 Interacts with YBX1 to Transcriptionally Regulate PD‐L1 and CD47, and Regulates the Antitumor Immune Response of CD8^+^ T Cells and Phagocytosis of Macrophages

**Figure 5:**
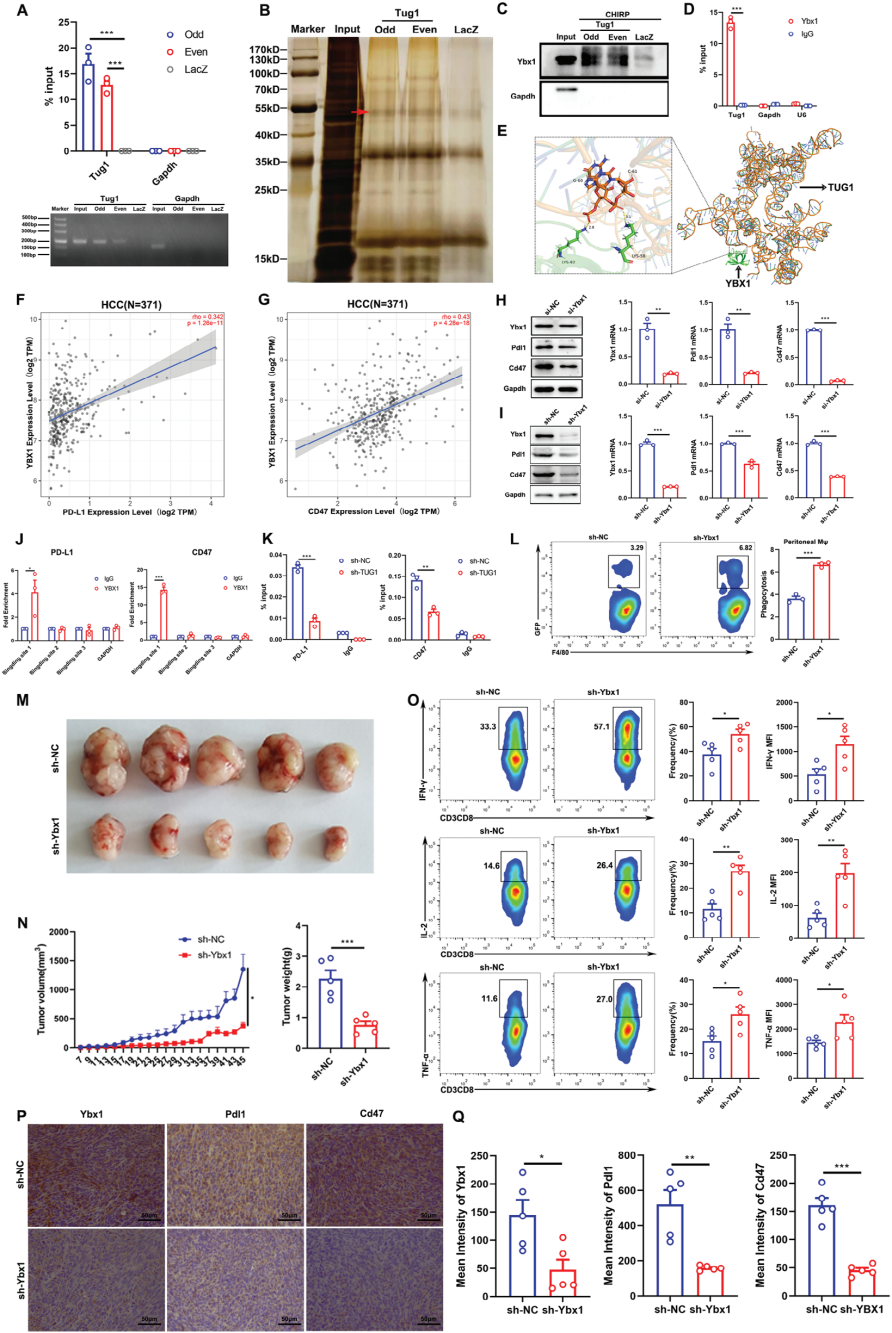
Tug1 interacts with Ybx1 to transcriptionally regulate Pdl1 and Cd47, thereby regulating the antitumor immune response of CD8^+^ T cells and phagocytosis of macrophages. A) Hepa1‐6 cells were used for CHIRP assay. The enrichment efficiency of Tug1 probes is confirmed via qPCR and electrophoresis. B) Silver staining of Tug1‐associated proteins from CHIRP. C) Enrichment of Ybx1 protein in the pull‐downs of both “odd” and “even” probes targeting Tug1 relative to LacZ probes. D) RIP‐qPCR shows enrichment of Tug1 after immunoprecipitation of Ybx1. E) The tertiary structure of TUG1 was docked with the YBX1 protein, the intermolecular interaction details were presented. F, G) The correlation of YBX1 with PD‐L1 and CD47 in HCC patients (*n* = 371). H) Pdl1 and Cd47 expressions in the control and Ybx1 siRNAs‐transfected Hepa1‐6 cells at protein and mRNA levels. I) Pdl1 and Cd47 expression in sh‐NC and sh‐Ybx1 Hepa1c1c7 cells at protein and mRNA levels. J) CHIP‐qPCR verifies the enrichment of YBX1 in the promoter regions of PD‐L1 and CD47. K) The CHIP‐qPCR results showed a reduction in the recruitment of YBX1 in the promoter regions of PD‐L1 and CD47 when TUG1 is downregulated. L) Representative plots and statistical analysis of macrophages phagocytosing sh‐NC or sh‐Ybx1 Hepa1‐6 cells (*n* = 3). M, N) Representative images of tumors, tumor growth curves, and tumor weights from sh‐NC and sh‐Ybx1 Hepa1‐6 cells tumor‐bearing mice (*n* = 5). O) The secretion of cytokines in CD8^+^ T cells from the tumors of sh‐NC and sh‐Ybx1 Hepa1‐6 cell‐bearing mice (*n* = 5). P, Q) The immunohistochemistry (IHC) staining and mean intensities of Ybx1, Pdl1 and Cd47. (*n* = 5. Magnification: 40 ×). Results are represented as the mean ± SEM. Statistical analysis was performed using the unpaired two‐tailed Student's t‐test. *, *p* < 0.05; **, *p* < 0.01; ***, *p* < 0.001.

Previous studies have reported that TUG1 has a bimodal distribution of fully spliced cytoplasmic and intron‐retained nuclear transcripts.^[^ ^22^ ^]^ TUG1 interacts with the PRC2 complex,^[^ ^23^ ^]^ localizes to PcG bodies, acts as a scaffold by interacting with Suv39h1‐methylated Pc2, and recruits growth control gene loci to PcG bodies.^[^ ^24^ ^]^ To further identify Tug1‐associated proteins that might be involved in the regulation of antitumor immune response, we performed chromatin isolation by RNA purification (ChIRP) with Hepa1‐6 cells following previously described,^[^ ^25^ ^]^ and confirmed the enrichment efficiency of the probes using qPCR and electrophoresis (**Figure** **5**A). The silver staining indicated a significantly stronger band slightly below 55KD compared to the control group (Figure 5B). Furthermore, we performed mass spectrometry, and the data obtained have been deposited into the ProteomeXchange Consortium via the iProX partner repository^[^ ^26^, ^27^ ^]^ with the dataset identifier “PXD044459”. We further found 290 proteins that interacted with Tug1, and the analysis of top GO terms for biological processes (BP), cellular component (CC), and molecular function (MF) are shown in Figure S8A (Supporting Information). Among the binding proteins, Ybx1 was one of the candidates that interacted with Tug1 with a high protein coverage, and the analysis of GO terms of Tug1‐related proteins related to Ybx1 showed that these proteins included RNA‐binding proteins and splicing factors (Figure S8B, Supporting Information). Furthermore, we performed CHIRP‐WB and confirmed that Ybx1 had significant interaction with Tug1 (Figure 5C). Then, we used RIP‐qPCR and confirmed that Ybx1 had significant interaction with Tug1 (Figure 5D). Considering that Tug1 may bind different chromatin regions in the nucleus, we predicted the molecular structure of Tug1 using the RNAfold web server (Figure S9A, Supporting Information). And then we predicted the interaction between Tug1 and Ybx1 using RPISeq and catRAPID. These bioinformatics results showed that Tug1 might directly bind to Ybx1 at a relevant high potential (Figure S9B–E, Supporting Information). Furthermore, we docked the tertiary structure of TUG1 with the YBX1 protein. Our analysis of the intermolecular interactions in the complex structure revealed 2 hydrogen bonds between the YBX1 protein and TUG1 nucleic acid. The first interaction is between the LYS‐58 residue of the YBX1 protein and the C‐61 of TUG1, with a bond length of 2.8 angstroms. The second interaction is between the LYS‐92 residue of the YBX1 protein and the G‐60 of TUG1, with a bond length of 3.1 angstroms (Figure 5E). We analyzed the protein‐nucleic acid docking free energy of the protein and lncRNA using Rosetta's Interface analyzer module, which was found to be −5.093 kcal mol^−1^. These results support the result that TUG1 can directly bind to YBX1.

As YBX1 also functions as a transcription factor apart from acting as an RNA‐binding protein,^[^ ^28^ ^]^ we examined whether TUG1 regulates PD‐L1 and CD47 by binding to YBX1. The positive correlation between YBX1 and PD‐L1, and between YBX1 and CD47 raise the possibility that YBX1 may function through interactions with PD‐L1 and CD47 (Figure 5F,G). We further investigated whether the TUG1‐YBX1 complex exerts its function through transcription regulation, and found that the downregulation of Ybx1 in Hepa1‐6 cells could decrease Pdl1 and Cd47 at both the protein and mRNA levels (Figure 5H). We also used another 2 siRNAs to confirm the regulation of Pdl1 and Cd47 by Ybx1, and the results showed that the downregulation of Ybx1 significantly decreased Pdl1 and Cd47 expressions at both the protein and mRNA levels (Figure S10A–D, Supporting Information). In addition, we confirmed the regulation of Pdl1 and Cd47 by Ybx1 in Hepa1c1c7 cells, and the results showed that the downregulation of Ybx1 significantly decreased Pdl1 and Cd47 expressions at both the protein and mRNA levels (Figure 5I). Then, the CHIP‐qPCR results indicated that YBX1 could bind to the promoters of PD‐L1 and CD47, and activate their transcription (Figure 5J). Furthermore, the CHIP‐qPCR results showed a significant reduction in the recruitment of YBX1 to the promoter regions of PD‐L1 and CD47 when TUG1 was knocked down (Figure 5K). These findings indicated that TUG1 is bound to YBX1 to facilitate its binding in the promoter regions of PD‐L1 and CD47, resulting in their transcriptional activation. In addition, the phagocytosis of Ybx1‐downregulation Hepa1‐6 cells by macrophages increased significantly (Figure 5L). The tumor volumes and tumor weights decreased significantly in sh‐Ybx1 Hepa1‐6 cell‐bearing mice (Figure 5M,N). Furthermore, CD8^+^ T cells displayed stronger activation phenotypes with more production of IFN‐γ, IL‐2, and TNF‐α (Figure 5O). Similarly, the secretions of IFN‐γ, IL‐2, and TNF‐α from CD4^+^ T cells clearly increased in sh‐Ybx1 Hepa1‐6 cell‐bearing mice (Figure S11A–F, Supporting Information). In addition, the IHC results showed that Ybx1, Pdl1, and Cd47 expressions decreased in tumors from sh‐Ybx1 Hepa1‐6 cell‐bearing mice (Figure 5P,Q). Furthermore, we also co‐cultured isolated CD8^+^ T cells with sh‐NC or sh‐Ybx1 Hepa1c1c7 cells, and the secretions of TNF‐α and IFN‐γ were increased in CD8^+^ T cells co‐cultured with sh‐Ybx1 Hepa1c1c7 cells (Figure S12A–D, Supporting Information). Then, we co‐cultured peritoneal cavity‐derived macrophages with sh‐NC or sh‐Ybx1 Hepa1c1c7 cells, and the phagocytosis of sh‐Ybx1 Hepa1c1c7 cells by peritoneal cavity‐derived macrophages increased significantly (Figure S12E,F, Supporting Information). These results indicated that the knockdown of Ybx1 in Hepa1c1c7 cells could restore the activation of CD8^+^ T cells, and increase the phagocytosis of cancer cells by macrophages. Taken together, Tug1 enhances the recruitment of Ybx1 to the promoter regions of Pdl1 and Cd47 to promote their expression, and then inhibits the function of CD8^+^ T cell and the phagocytosis function of macrophages toward cancer cells, thereby regulating the antitumor immune response.

### 2.7 TUG1 is a Potential Biomarker and Immunotherapeutic Target for Liver Cancer

**Figure 6:**
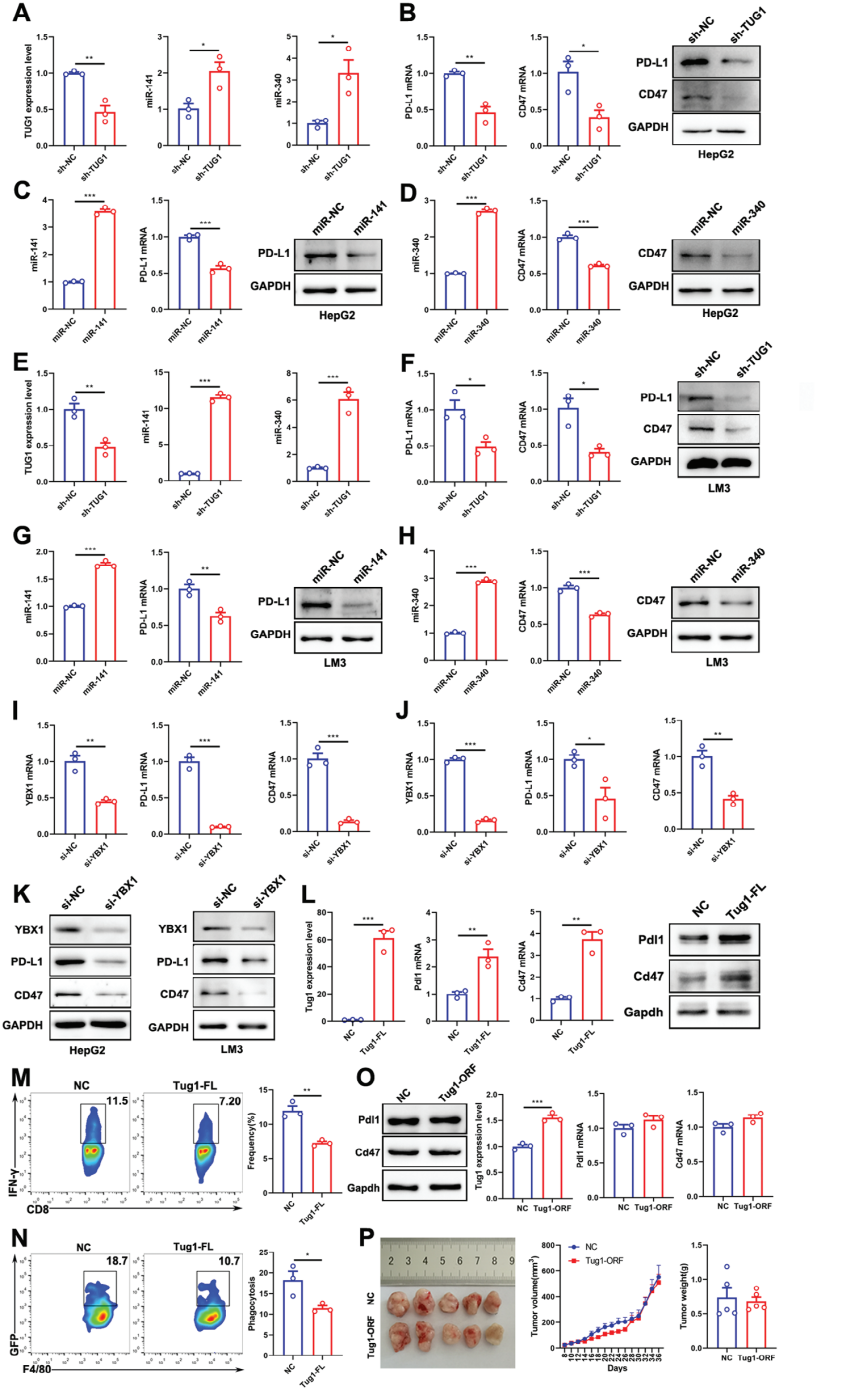
TUG1, but not TUG1‐ORF, regulates the expressions of PD‐L1 and CD47 in liver cancer cells. A) The expressions of miR‐141 and miR‐340 are upregulated in TUG1‐downregulated HepG2 cells. B) PD‐L1 and CD47 expressions at both the mRNAs and protein levels in TUG1‐downregulated HepG2 cells. C) The expression of miR‐141 and PD‐L1 in the control and miR‐141 over‐expressed HepG2 cells. D) The expressions of miR‐340 and CD47 in the control and miR‐340 over‐expressed HepG2 cells. E) The expressions of miR‐141 and miR‐340 are upregulated in TUG1‐downregulated LM3 cells. F) PD‐L1 and CD47 expressions at both the mRNAs and protein levels in TUG1‐downregulated LM3 cells. G) The expression of miR‐141 and PD‐L1 in the control and miR‐141 over expressed LM3 cells. H) The expressions of miR‐340 and CD47 in the control and miR‐340 over expressed LM3 cells. I) PD‐L1 and CD47 expressions at mRNA levels in YBX1‐downregulated HepG2 cells. J) PD‐L1 and CD47 expressions at mRNA levels in YBX1‐downregulated LM3 cells. K) PD‐L1 and CD47 expressions at protein levels in YBX1‐downregulated HepG2 cells and LM3 cells. L) Pdl1 and Cd47 expressions at both the mRNAs and protein levels in Tug1 full length (Tug1‐FL)‐overexpressed Hepa1‐6 cells. M) The frequencies of IFN‐γ in CD8^+^ T cells co‐cultured with NC and Tug1‐FL Hepa1‐6 cells (*n* = 3). N) Representative plots and statistical analysis of macrophages phagocytosing NC or Tug1‐FL Hepa1‐6 cells (*n* = 3). O) Pdl1 and Cd47 expressions at both the mRNAs and protein levels in Tug1‐ORF‐overexpressed Hepa1‐6 cells. P) Representative images of tumors, tumor growth curves, and tumor weights from mice bearing NC and Tug1‐ORF‐overexpressed Hepa1‐6 cells tumor‐bearing mice (*n* = 5). Results are represented as the mean ± SEM. Statistical analysis was performed using the unpaired two‐tailed Student's t‐test.*, *p* < 0.05; **, *p* < 0.01; ***, *p* < 0.001.

To further confirm the mechanism of TUG1 in regulating antitumor immunity, we downregulated TUG1 in human liver cancer HepG2 cells and found that both miR‐340 and miR‐141 were upregulated in TUG1‐downregulated cells (**Figure** **6**A), while the expressions of PD‐L1 and CD47 decreased (Figure 6B). We also transfected miR‐340 and miR‐141 into HepG2 cells, respectively. The results showed that over expression of miR‐141 decreased the expression of PD‐L1 (Figure 6C), and over expression of miR‐340 decreased the expression of CD47 in HepG2 cells (Figure 6D). In addition, we downregulated TUG1 in another human liver cancer LM3 cells and found that both miR‐340 and miR‐141 were upregulated in TUG1‐downregulated cells (Figure 6E), while the expressions of PD‐L1 and CD47 decreased (Figure 6F). We also transfected miR‐340 and miR‐141 into LM3 cells, respectively. The results showed that overexpression of miR‐141 decreased the expression of PD‐L1 (Figure 6G), and over expression of miR‐340 decreased the expression of CD47 levels in LM3 cells (Figure 6H). These results validated that TUG1 regulated PD‐L1 and CD47 by acting, respectively, as a sponge of miR‐141 and miR‐340. To confirm the regulation of PD‐L1 and CD47 by YBX1 in human liver cancer cells, we knocked down YBX1 in HepG2 cells and LM3 cells, respectively. The results showed that the mRNA level of PD‐L1 and CD47 decreased both in HepG2 cells and LM3 cells when YBX1 was knocked down (Figure 6I,J), as well as the protein levels (Figure 6K). These results verified the transcription regulation of PD‐L1 and CD47 by TUG1‐YBX1 complex.

Furthermore, we overexpressed the full length of Tug1(Tug1‐FL) in Hepa1‐6 cells to evaluate the regulation of Tug1 to Pdl1 and Cd47. The results showed that overexpression of Tug1‐FL increased the expressions of Pdl1 and Cd47 at both the mRNA and protein levels (Figure 6L). We also co‐cultured isolated CD8^+^ T cells with NC or Tug1‐FL‐overexpressed Hepa1‐6 cells, and the secretion of IFN‐γ was decreased in CD8^+^ T cells co‐cultured with Tug1‐FL‐overexpressed Hepa1‐6 cells (Figure 6M). Then, we co‐cultured peritoneal cavity‐derived macrophages with NC or Tug1‐FL‐overexpressed Hepa1‐6 cells, and the phagocytosis of Tug1‐FL‐overexpressed Hepa1‐6 cells by peritoneal cavity‐derived macrophages decreased significantly (Figure 6N). These results indicated that an over expression of Tug1‐FL could inhibit the activation of CD8^+^ T cells and decreased the phagocytosis of cancer cells by macrophages. Studies show that lncRNA TUG1 has a very complex mechanism of function, being localized in the nucleus and in the cytoplasm. It also has a highly conserved non‐canonical translation initiation codon (CUG) ORF and could be translated into a microprotein of 153aa in size.^[^ ^18^, ^29^ ^]^ To explore whether TUG1 plays a role in tumor immune regulation by small proteins, we transferred Tug1‐ORF into Hepa1‐6 cells, and the results showed that neither Pdl1 nor Cd47 changed at the protein and mRNA levels in Tug1‐ORF‐overexpressed cells (Figure 6O), the overexpression of Tug1‐ORF did not affect tumor growth (Figure 6P). We also transferred TUG1‐ORF into HepG2 cells; similarly, the results showed that neither PD‐L1 nor CD47 changed at the protein and mRNA levels in TUG1‐ORF‐overexpressed cells (Figure S13A,B, Supporting Information). Furthermore, we co‐cultured isolated CD8^+^ T cells with NC or Tug1‐ORF Hepa1‐6 cells, and found that the secretion of TNF‐α, IFN‐γ and IL‐2 did not change (Figure S13C–E, Supporting Information). We also co‐cultured peritoneal cavity‐derived macrophages with NC or Tug1‐ORF Hepa1‐6 cells, and found that the phagocytosis of macrophages did not change (Figure S13F,G, Supporting Information). These results indicated that Tug1‐ORF does not play a critical role in antitumor immunity.

**Figure 7:**
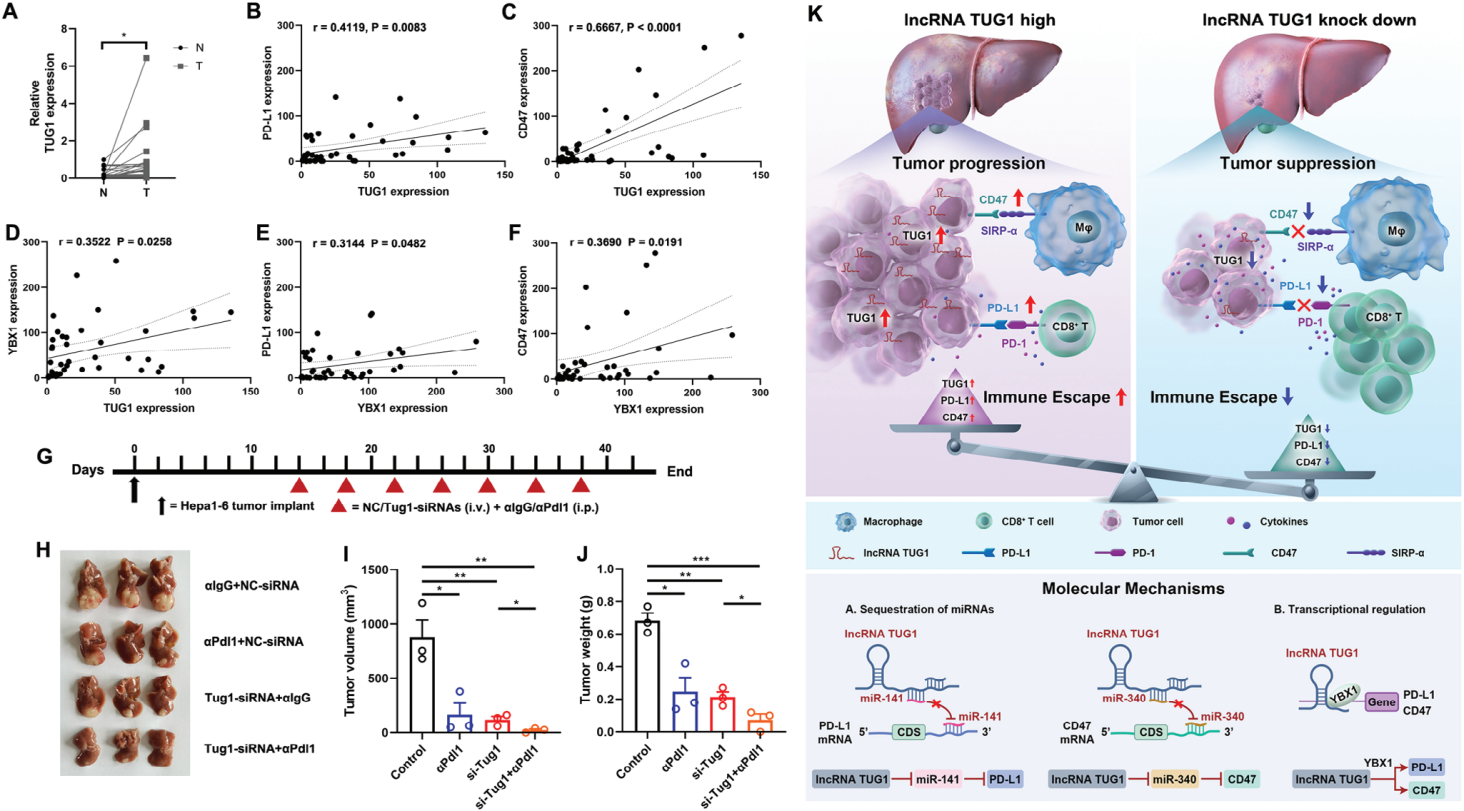
TUG1 is a potential biomarker and immunotherapeutic target for liver cancer. A) Expression of TUG1 in 40 pairs of clinical specimens of both normal and tumor tissues was determined by qRT‐PCR. B) Correlative analysis of the relative expression of TUG1 with PD‐L1 in human HCC tumors (*n* = 40). C) Correlative analysis of the relative expression of TUG1 with CD47 in human HCC tumors (*n* = 40). D) Correlative analysis of the relative expression of TUG1 with YBX1 in human HCC tumors (*n* = 40). E) Correlative analysis of the relative expression of YBX1 with PD‐L1 in human HCC tumors (*n* = 40). F) Correlative analysis of the relative expression of YBX1 with CD47 in human HCC tumors (*n* = 40). G) Schematic of Tug1 siRNAs therapy in combination with an anti‐Pdl1 antibody. H–J) Representative images of tumors, tumor volumes, and tumor weights (*n* = 3). K) Schematic of the molecular mechanism of TUG1 in regulating antitumor immune response. Results are represented as the mean ± SEM. Statistical analysis was performed using the unpaired two‐tailed Student's t‐test. *, *p* < 0.05; **, *p* < 0.01; ***, *p* < 0.001.

To determine the levels of TUG1 in human HCC clinical samples, we examined their expression levels in 40 pairs of human HCC tissues and matched normal adjacent tissues using qRT‐PCR. Indeed, the human HCC tissues displayed significantly increased expression of TUG1 compared to the matched normal adjacent tissues (**Figure** **7**A). Moreover, TUG1 expression positively correlated with PD‐L1, CD47, and YBX1 expressions (Figure 7B–D), while YBX1 expression positively correlated with PD‐L1 and CD47 expressions (Figure 7E,F).

To investigate the antitumor effect of a combination of knockdown of Tug1 with the use of a Pdl1 monoclonal antibody, orthotopic HCC mouse models were randomly divided into 4 groups and treated with IgG plus NC‐siRNAs, anti‐Pdl1 plus NC‐siRNAs, IgG plus Tug1‐siRNAs, or anti‐Pdl1 plus Tug1‐siRNAs separately (Figure 7G). We found that either anti‐Pdl1 or Tug1‐siRNAs treatment partially inhibited tumor growth, and the combination had a synergistic effect on either tumor volumes (Figure 7H‐I) and tumor weights (Figure 7J), which confirmed that Tug1 could act as a potential therapeutic target to improve the therapeutic efficacy of Pdl1 checkpoint blockades. Taken together, TUG1 upregulate PD‐L1 to inhibit the antitumor immune response of CD8^+^ T cells, and upregulates CD47 to inhibit the phagocytosis of macrophages, ultimately promoting tumor immune escape. The down‐regulation of TUG1 in HCC cells can inhibit both PD‐L1 and CD47, which indicates that TUG1 is a potential biomarker and immunotherapeutic target for liver cancer, thereby providing new ideas for enhancing antitumor immunity (Figure 7K).

## 3 Discussion

HCC is a typical inflammation‐associated tumor that usually develops due to an immunosuppressive status, making immunotherapy an attractive therapeutic strategy.^[^ ^30^ ^]^ Immune checkpoint blockades (ICBs) induce an immune response against tumors, and these strategies have been implemented in the clinical setting with encouraging therapeutic effects.^[^ ^31^, ^32^ ^]^ However, a low objective response rate, as well as potential systemic side effects, brings great challenges.^[^ ^33^ ^]^ Additionally, the storage and distribution of PD‐L1 in cells can minimize the therapeutic responses of ICB‐based therapies.^[^ ^34^ ^]^ Recently, various therapeutic strategies to modulate macrophage phagocytosis against tumor cells have been proposed, and anti‐CD47 antibodies have been reported to be able to suppress tumor growth and enhance the effects of chemotherapy in HCC.^[^ ^35^ ^]^ However, further clinical translation has been limited by side effects.^[^ ^36^ ^]^ Recent studies reported that PD‐L1 and CD47 coordinate to promote tumor immune evasion,^[^ ^14^ ^]^ and dual targeting of PD‐L1 and CD47 in tumor cells using a bispecific antibody significantly limits immune evasion.^[^ ^37^ ^]^ However, due to the complexity of the tumor immune microenvironment and the diversity of immunosuppressive mechanisms, the effectiveness of antibody‐based drugs is still limited. The microanatomical structure of the liver allows hepatocytes to be easily transduced or transfected in vivo, and therapeutic approaches that directly target lncRNAs, including siRNAs, antisense oligonucleotides (ASO), and aptamers, or delivery tumor‐suppressive lncRNAs to target cells via nanoparticles or exosomes could help improve HCC treatment.^[^ ^38^ ^]^ Accumulating evidence suggests that abnormal expression of lncRNAs is closely associated with tumor immunity.^[^ ^15^ ^]^ The long non‐coding RNA TUG1 is upregulated in numerous cancers including HCC, and the cancer biological functions of TUG1 have been well studied.^[^ ^19^, ^20^ ^]^ However, how TUG1 is upregulated in cancers and the effects of its upregulation on the tumor immune microenvironments are incompletely understood. To further improve the clinical effect of current immunotherapy in HCC, it is urgent to elucidate the molecular role and mechanism of TUG1 in regulating critical immune cells in HCC.

N6‐Methyladenosine (m^6^A) is one of the most universal RNA modifications among various post‐transcriptional modifications identified in mRNAs and non‐coding RNAs.^[^ ^39^ ^]^ Recent studies have shown that m^6^A modification plays a critical role in the regulation of lncRNAs in cancers.^[^ ^40^ ^]^ In this study, we found that METTL3 mediated m^6^A modification‐induced upregulation of TUG1, which negatively correlated with the prognosis of HCC patients. The knockdown of Tug1 significantly inhibited tumor growth and metastasis. We further explored the effects of Tug1 upregulation on the tumor immune microenvironments via flow cytometry and found that CD8^+^ T cells significantly increased and displayed stronger activation phenotypes with more production of IFN‐γ, IL‐2, and TNF‐α in sh‐Tug1 Hepa1‐6 cell‐bearing mice. Moreover, M1‐like macrophages in the spleens and tumors of Tug1‐knockdown tumor‐bearing mice clearly increased. In addition, the CD8^+^ T cells in the sh‐Tug1 Hepa1c1c7 cell‐bearing mice also displayed stronger activation phenotypes with more production of IFN‐γ and TNF‐α, and the M1‐like macrophages in the spleens and tumors also clearly increased, which indicated that m^6^A‐mediated upregulation of TUG1 may be closely related to tumor immunity.

The complex interactions of several cellular populations have an important influence on tumor immune evasion.^[^ ^41^ ^]^ CD8^+^ T cells play critical roles in selectively detecting and eradicating cancer cells. The tumor immune microenvironment exhibits dysfunctional CD8^ +^ T cells with a decreased ability to proliferate and release effector cytokines.^[^ ^42^ ^]^ Inhibitory receptors, such as PD‐1, T‐cell immunoglobulin, and mucin‐domain containing‐3 (TIM3), are highly expressed in exhausted CD8 ^+^ T cells.^[^ ^43^ ^]^ In activated T lymphocytes, PD‐1 is upregulated and inhibits T‐cell function by binding to B7‐H1 (also known as PD‐L1, and CD274), as well as B7‐DC (also known as PD‐L2 and CD273).^[^ ^44^ ^]^ In addition, tumor‐associated macrophages (TAMs) can differentiate into different functional phenotypes in the tumor immune microenvironment: the M1‐like subtype plays a substantial role in antigen presentation, while M2‐like TAMs exert pro‐tumorigenic activities.^[^ ^45^ ^]^ A previous study reported that treatment targeted to CD47 stimulates phagocytosis of tumor cells by macrophages and promotes M1‐type polarization of macrophages.^[^ ^46^ ^]^ We further found that TUG1 and METTL3 were positively correlated with 2 immune checkpoints: PD‐L1 and CD47. The knockdown of METTL3 significantly decreased PD‐L1 and CD47 expressions, which indicates that m^6^A‐mediated upregulation of TUG1 is closely related to tumor immunity and may mediate tumor immune escape by regulating immune checkpoints, which can serve as novel immunotherapeutic strategies for clinical patients suffering from HCC.

As the interactions between PD‐L1 and PD‐1 can inhibit the effector functions of tumor‐infiltrating T cells,^[^ ^47^ ^]^ we further elucidated that the knockdown of Tug1 in tumor cells promoted the co‐cultivated CD8^+^ T cells to display stronger activation phenotypes. Additionally, as the phagocytic effect of macrophages on tumor cells was enhanced after blocking CD47, we explored and found that the knockdown of Tug1 in tumor cells dramatically enhanced the phagocytosis of co‐cultivated macrophages. While overexpression of tug1 exhibited an opposite effect. Mechanistically, TUG1 sponges miR‐141 promote PD‐L1 expression and regulate the antitumor immune response of CD8^+^ T cells. Meanwhile, TUG1 directly interacts with miR‐340 to promote CD47 and the phagocytosis of macrophages. Moreover, TUG1 interacts with YBX1 to facilitate the upregulation of PD‐L1 and CD47 transcriptionally, thereby, regulating the antitumor immune response of CD8^+^ T cells and the phagocytosis of macrophages. As TUG1 localizes in both the nucleus and the cytoplasm, it also has a highly conserved non‐canonical translation initiation codon (CUG) ORF and could be translated into a microprotein of 153aa in size.^[^ ^18^, ^29^ ^]^ We further performed experiments to explore whether TUG1 plays a role in tumor immune regulation by small proteins. Both in vitro and in vivo results indicated that TUG1‐ORF does not play a critical role in antitumor immunity. Considering that TUG1 may bind to different chromatin regions in the nucleus, we performed chromatin isolation by RNA purification (ChIRP) and RIP, which confirmed that Tug1 interacted with Ybx1. We further predicted the molecular structure of Tug1 using the RNAfold web server and then predicted the interaction between Tug1 and Ybx1 using RPISeq and catRAPID, which showed that Tug1 might directly bind to Ybx1 at a high relevance. Furthermore, we docked the tertiary structure of TUG1 with the YBX1 protein, the results found the first interaction is between the LYS‐58 residue of the YBX1 protein and the C‐61 of TUG1, the second interaction is between the LYS‐92 residue of the YBX1 protein and the G‐60 of TUG1. These results support that TUG1 could directly bind to YBX1.

To determine the levels of TUG1 in human HCC clinical samples, we examined their expression levels in 40 pairs of human HCC tissues and matched normal adjacent tissues. The results showed that the human HCC tissues displayed significantly increased expression of TUG1 compared to the matched normal adjacent tissues. Moreover, TUG1 expression positively correlated with PD‐L1, CD47, and YBX1, while YBX1 expression positively correlated with PD‐L1 and CD47. Overall, we elucidated a new mechanism underlying how TUG1 regulates tumor immunity. Our study also demonstrated that combining Tug1‐siRNAs with Pdl1 checkpoint blockaders could enhance the antitumor response and offer a novel therapy to be tested in clinical trials with HCC patients.

## 4 Conclusion

In summary, our results demonstrate that TUG1 regulates PD‐L1 and CD47 in HCC, which further inhibits CD8^+^ T cells activation and phagocytosis of macrophages, thereby regulating tumor immune escape. Mechanistically, TUG1 regulates PD‐L1 and CD47 expressions by acting as a sponge of miR‐141 and miR‐340, respectively. Meanwhile, TUG1 interacts with YBX1 to facilitate the upregulation of PD‐L1 and CD47 transcriptionally. Clinically, TUG1 positively correlates with PD‐L1 and CD47 in HCC tissues. Moreover, a combination of Tug1‐siRNA therapy with a Pdl1 antibody effectively suppresses tumor growth. Therefore, our study highlights the great potential of inhibiting TUG1 to enhance patient response to immunotherapy, which may provide a complementary immunotherapeutic approach for HCC patients.

## 5 Experimental Section

### Cell Culture

Hepa1‐6, HepG2, Hepa1c1c7, LM3, and 293T cells were originally obtained from the American Type Culture Collection (ATCC, USA), and cultured with Dulbecco´s modified Eagles medium (DMEM, Gibco, USA) supplemented with 10% fetal bovine serum (FBS, Gibco, USA), 100 U mL^−1^ penicillin and 100 µg mL^−1^ streptomycin (solarbio, China) in a cell culture incubator containing 5% CO_2_ at 37 °C.

### Patients and Clinical Tissue Specimens

Human HCC tissues were obtained from the First Affiliated Hospital of Guangdong Pharmaceutical University (Guang Zhou, China). The total RNA of the fresh‐frozen materials was isolated using TRIzol reagent (Invitrogen, USA). The study was approved by the Institutional Research Ethics Committee of the First Affiliated Hospital of Guangdong Pharmaceutical University.

### Generation of Macrophages and In Vitro Phagocytosis Assay

The phagocytosis assays were performed following previously described procedures.^[^ ^48^ ^]^ In brief, the bone marrow‐derived macrophages were obtained from the femur and tibia of C57BL/6 mice and cultured with RPMI‐1640 (Gibco, USA) complete medium added with recombinant mouse M‐CSF (20 ng mL^−1^, PeproTech, USA) for 5 days. To obtain macrophages obtained from the peritoneal cavity, a 3% thioglycolate solution was injected intraperitoneally, and the thioglycolate‐elicited peritoneal macrophages were harvested 3 days later. After 2 h, nonadherent cells were removed. Next, 5 × 10^4^ bone marrow‐derived or peritoneal‐derived macrophages were separately seeded for 24 h, and then co‐cultivated with 2 × 10^4^ GFP^+^ cancer cells for 4 h. Subsequently, the cells were stained with an anti‐mouse F4/80 antibody (Biolegend, USA) and analyzed using a FACS Celesta flow cytometer (BD Biosciences, USA). For the immunofluorescence assay, the slides were incubated with the F4/80 antibody (Bio‐rad, #MCA497GA, 1:200) at 4  °C overnight. On the next day, the slides were incubated with Alexa Fluor 555‐conjugated goat anti‐rat IgG (CST, 1:500) in the dark for 1 h. Then, the 4′,6‐diamidino‐2‐phenylindole (DAPI) was used to identify the nuclei. Immunofluorescence images were acquired using a Laser Scanning Confocal Microscope (OLYMPUS FV3000, Japan).

### In Vitro Co‐Cultivation of Tumor Cells with CD8^+^ T Cells

CD8^+^ T cells were isolated using CD8α microbeads (Miltenyi Biotec, Germany), followed by activation by bound anti‐CD3 (5 µg mL^−1^; BD Biosciences, USA) and anti‐CD28 (2 µg mL^−1^; BD Biosciences, USA) in a 96‐well plate. Next, 2 × 10^5^ CD8^+^ T cells were co‐cultivated with cancer cells at a ratio of 10:1 for 48 h. Then, the cells were stimulated with a cell stimulation cocktail plus protein transport inhibitors (Invitrogen, USA) for 4 h at 37 °C. The co‐cultured cells were collected and the secretion of intracellular cytokines in CD8^+^ T cells was determined via flow cytometry.

### Quantitative Real‐Time PCR

TRIzol reagent (Invitrogen, USA) was used to extract total RNA according to the manufacturer's instructions. PrimeScript RT Master Mix (Takara Bio, China) was used to perform reverse transcription with 1 µg of total RNA. TB Green Premix Ex Taq II (Takara Bio, China) was used for quantitative real‐time PCR (qRT‐PCR), and each reaction was performed using an ABI StepOneTM Real‐Time PCR System (Applied Biosystems Inc., USA). GAPDH was used as an internal reference for genes, and U6 was used as an internal reference for miRNAs. The comparative cycle threshold method by 2^−ΔΔCt^ was used to determine the fold changes. The primers used for the real‐time PCR are shown in Table S1 (Supporting Information).

### Western Blotting

Whole‐cell lysates were prepared using RIPA lysis buffer (Beyotime Biotechnology, China) in the presence of protease inhibitors, and quantified with a BCA Assay Kit (Beyotime Biotechnology, China). Proteins were electrophoresed using 10% SDS‐PAGE gel and were then transferred to PVDF membranes and blocked with 5% skim milk. The membranes were incubated in diluted primary antibodies at 4 °C overnight. The immunoblots were examined using an ECL detection system (Millipore, USA). The antibodies used are listed in Table S2 (Supporting Information).

### Chromatin Isolation by RNA Purification (ChIRP)

The ChIRP Kit (BersinBio, Guangzhou, China) was used to perform the ChIRP assay according to the manufacturer's instructions. Briefly, 8 × 10^7^ Hepa1‐6 cells were prepared, and 1% formaldehyde was used to crosslink the cells for 10 min, which were quenched using 1.375 m glycine for 5 min. Then, the cells were lysed with swelling buffer supplemented protease inhibitor and DTT, homogenized the sample using a homogenizer on ice, then centrifuged at 2500 g for 5 min at 4 °C to collect the cell pellet, fully resuspend the cell pellet by adding nuclear lysis buffer containing protease inhibitor, DTT and RNase Inhibitor. Then stand on ice for 10 min and sonicated using a Bioruptor (Scientz, China) at 4 °C on the high setting with pulse intervals of 3 s on and 5 s off for a total of 20 min to obtain ≈200–500 bp DNA fragments. The Tug1: chromatin complex was captured using a pool of biotin probes with specific recognition for Tug1. The probes were separated into 2 pools: the “odd” pool included the probes PC689, PC691, PC693, etc, and the “even” pool included the probes PC688, PC690, PC692, etc. The control probe targeting LacZ mRNA was used as a negative control. The ChIRP probes sequences are listed in Table S3 (Supporting Information). The cell lysates were incubated with the biotinylated probe overnight at 37 °C in a Hybridizer Oven (UVP HB‐1000, USA). The binding complex was covered with streptavidin‐conjugated magnetic beads. RNA extraction was performed using TRIzol reagent to confirm the RNA enrichment. Protein elution was performed by resuspending the beads in elution buffer added with DTT at 37 °C for 2 h. The proteins were analyzed by liquid chromatograph mass spectrometry (LC–MS) and detected using western blotting.

### Chromatin Immunoprecipitation Assay (CHIP)

CHIP was performed following previously described procedures.^[^ ^49^ ^]^ In brief, 4 × 10^7^ cells were prepared, and 1% formaldehyde was used to fix the cells for 10 min, which were then quenched using glycine at a final concentration of 125 mM for 5 min. The cells were washed twice in cold PBS and scraped. The cells were resuspended in a CHIP lysis buffer (50 mmol L^−1^ Tris‐HCl, pH 8.0, 5 mmol/L EDTA, 1% SDS supplemented with protease inhibitors). Then, the cells were sonicated to a size range of 200–500 bp using a Bioruptor Sonicator. The chromatin fraction was immunoprecipitated with the corresponding antibodies overnight at 4 °C. The RNase A was used to treat the samples at 37 °C for 30 min after elution and reversal cross‐linking was carried out overnight at 65 °C. The purified DNA was analyzed via qPCR using the indicated primers (Table S4, Supporting Information).

### RNA Immunoprecipitation (RIP)

The RIP kit (BersinBio, Guangzhou, China) was used to perform the RIP assay in accordance with the manufacturer's protocol. Briefly, 4 × 10^7^ cells were lysed in a polysome lysis buffer supplemented with protease inhibitors and RNase inhibitors, and incubated with a RIP buffer containing magnetic beads conjugated with 4 µg anti‐YBX1 antibodies (Proteintech) or immunoglobulin G (IgG) antibodies (BersinBio, Guangzhou) at 4 °C overnight. Then, proteins were digested by proteinase K, and co‐immunoprecipitated RNAs were eluted and analyzed using qRT‐PCR.

### M^6^A RNA Immunoprecipitation (MeRIP)

The MeRIP Kit (BersinBio, Guangzhou, China) was used to perform the MeRIP assay according to the manufacturer's instructions. In brief, 4 × 10^7^ cells were prepared and washed twice in cold PBS, then centrifuged to collect the cell pellet. Total RNA was extracted with TRIzol reagent and fragmented into ≈300 nt using a fragmentation buffer at 94 °C for 5 min, and 50 µL was separated as input. The remaining samples were divided into 2 groups and incubated with m^6^A or IgG antibodies (BersinBio, Guangzhou) at 4 °C for 4 h. Then treated with protein A/G magnetic beads at 4 °C for an additional 1 h. Subsequently, the beads were washed and resuspended in an elution buffer. After rotation and elution, the RNA enrichment was purified via Phenol/Chloroform/Isoamylalcohol (25:24:1, Solarbio) extraction, and then analyzed using qRT‐PCR.

### Animals and In Vivo Tumorigenesis Assay

For this assay, the 6–8 weeks‐old female C57BL/6 mice were purchased from the Guangdong Medical Laboratory Animal Center (Guangzhou, China). The care and treatment of these mice were approved by the animal ethics committee of Guangdong Pharmaceutical University (Guangzhou, China).

For the subcutaneous model of HCC, 2 × 10^6^ cancer cells were subcutaneously injected into the right dorsal part of C57BL/6 mice. The length (L) and width (W) of tumors were monitored every other day. Tumor size was calculated using the following equation: (L × W^2^)/2. For the orthotopic mouse model of HCC, 2 × 10^6^ cancer cells were injected into the right dorsal part of the mice to form subcutaneous tumors, and the mice were euthanized when their subcutaneous tumors grew to a length of 1 cm. The tumors were peeled and cut into ≈1 mm^3^ pieces under aseptic conditions. Then, a single tumor piece was inoculated into the liver parenchyma of the left lobe of recipient mice under anesthesia. For the lung metastasis mouse model, 1 × 10^6^ cancer cells were injected into the tail vein of the mice, and then the mice were euthanized 5 weeks after injection and the metastatic nodules in the lungs were counted. For the combination treatment, an orthotopic mouse model of HCC was established with Hepa1‐6 cells. The mice were randomly divided into 4 groups according to the dosing regimens described in the Results section. From day 12, the siRNAs were administered intravenously at a total volume of 10 nmol/mouse. Anti‐ Pdl1 antibodies (Clone No.10F.9G2, BioXcell) or IgG isotype control were given intraperitoneally at 200 µg/day every 4 days. All mice were euthanized after treatment at the indicated time.

### Flow Cytometry and Intracellular Staining

For the detection of mouse macrophages, the cells were stained with a combination of APC‐cy7‐CD45, BV605‐Ly6G, PE‐CD11b, APC‐F4/80 and PE‐cy7‐MHC II (Biolegend, USA) on ice for 30 min. For the detection of lymphocytes, single cells of the spleens or tumors were stained with APC‐cy7‐CD45, PE‐cy7‐CD3, Percp‐cy5.5‐CD4, and APC‐CD8α (Biolegend, USA) on ice for 30 min. Then, the cells were detected using a FACSCelesta flow cytometer (BD Biosciences, USA). All data were analyzed using the software FlowJo (Tree Star Inc., Ashland, OR, USA).

For intracellular staining of cytokines, T lymphocytes derived from the spleens and tumors of tumor‐bearing mice were incubated in an RPMI‐1640 complete medium supplemented with 200 nM of L‐glutamine and 0.05 mM of β‐mercaptoethanol. After being stimulated with a cell stimulation cocktail (plus protein transport inhibitors, Invitrogen, USA) for 4 h, the cells were stained with APC‐cy7‐CD8α and BV421‐ CD3 (Biolegend, USA). Then, the cells were fixed with an IC fixation buffer for 30 min at room temperature and permeabilized with a permeabilization solution (Invitrogen, USA). Finally, T cells were further stained with PE‐IFN‐γ, APC‐TNF‐α and PE‐cy7‐IL‐2, and analyzed by a flow cytometer.

### Bioinformatics Analysis

The expression of TUG1 in the HCC samples and normal samples were compared with data from The Cancer Genome Atlas (TCGA) database; The expression profiles of HBV‐HCCs, HCV‐HCCs, non‐viral‐HCCs, NAFLD, and healthy control were obtained from the GSE107170, GSE87630, GSE14323, GSE190967 and GSE159088 datasets; The effect of TUG1 on the overall survival of HCC patients was analyzed using the Kaplan‐Meier Plotter (http://kmplot.com/analysis/).^[^ ^50^ ^]^ Clinical relevance was analyzed using the Tumor IMmune Estimation Resource (TIMER, http://timer.cistrome.org/).^[^ ^51^ ^]^ The potential binding sites of miRNAs with the targeted genes were predicted by using the online bioinformation database ENCORI/starBase (https://rnasysu.com/encori/).^[^ ^52^ ^]^ The potential binding sites of miRNAs with TUG1 were predicted by using DIANA‐LncBase (https://diana.e‐ce.uth.gr/lncbasev3).^[^ ^53^ ^]^ The molecular structure of TUG1 was predicted using the RNAfold web server (http://rna.tbi.univie.ac.at/cgi‐bin/RNAWebSuite/RNAfold.cgi), and the interaction between Tug1 and Ybx1 was predicted using RPISeq (http://pridb.gdcb.iastate.edu/RPISeq/)^[^ ^54^ ^]^ and catRAPID (http://service.tartaglialab.com/page/catrapid_group).^[^ ^55^ ^]^

### Molecular Docking

The resolved structure of the protein YBX1 was obtained from the RCSB PDB website (https://www.rcsb.org/) (PDB ID: 6KUG),^[^ ^56^ ^]^ and the protein was prepared using Pymol software for hydrogenation, dehydration, and other preparations. Then, we used 3dRNA for lncRNA modeling (http://biophy.hust.edu.cn/new/3dRNA).^[^ ^57^ ^]^ Rigid docking was performed using HADDOCK to dock the tertiary structure of lncRNA TUG1 with the YBX1 protein. The resulting output was analyzed using the force analysis module of Chimera, and 3D conformation and force display were performed using Pymol.

### Statistical Analysis

All data were analyzed using GraphPad Prism 8.0 and performed using a two‐tailed unpaired Student's t‐test or two‐way analysis of variance (two‐way ANOVA). Spearman correlation analysis was used to determine the relationship between the indicated genes. The data were presented as the mean ± SEM (standard error of the mean) of triplicate measurements in a representative experiment. The statistical significance include: *p < 0.05; **p < 0.01; *** *p* < 0.001.

## Conflict of Interest

The authors declare no conflict of interest.

## Author Contributions

Q.X., G.Y., X.H., and H.Z. contributed equally to this work. Q.X. and R.Z. conceived and designed the study; Q.X., G.Y., X.H., H.Z., L.L., B.L., L.W., and X.W. performed the experiments; C.F., Y.Y., Z.Y., H. Z., W.C., and Y.L. helped with animal experiments; Q.C. and H.S. helped with bioinformatic analysis. Q.X. and R.Z. analyzed and interpreted the data; Q.X. drafted the manuscript. Q.X., R.Z., and L.L. reviewed and revised the manuscript. R.Z., Q.X., and H.Z. acquired funding. All authors read and approved the final manuscript.

## Supporting information

Supporting Information

## Data Availability

The data that support the findings of this study are available from the corresponding author upon reasonable request.

## References

[1] H. Sung , J. Ferlay , R. L. Siegel , M. Laversanne , I. Soerjomataram , A. Jemal , F. Bray , Ca‐Cancer J. Clin. 2021, 71, 209. 33538338 10.3322/caac.21660

[2] J. D. Yang , P. Hainaut , G. J. Gores , A. Amadou , A. Plymoth , L. R. Roberts , Nat. Rev. Gastroenterol. Hepatol. 2019, 16, 589. 31439937 10.1038/s41575-019-0186-y PMC6813818

[3] P. Tabrizian , G. Jibara , B. Shrager , M. Schwartz , S. Roayaie , Ann. Surg. 2015, 261, 947. 25010665 10.1097/SLA.0000000000000710

[4] A. Forner , M. Reig , J. Bruix , Lancet 2018, 391, 1301. 29307467 10.1016/S0140-6736(18)30010-2

[5] A.‐L. Cheng , C. Hsu , S. L. Chan , S.‐P. Choo , M. Kudo , J. Hepatol. 2020, 72, 307. 31954494 10.1016/j.jhep.2019.09.025

[6] D. S. Chen , I. Mellman , Immunity 2013, 39, 1. 23890059 10.1016/j.immuni.2013.07.012

[7] S. H. Baumeister , G. J. Freeman , G. Dranoff , A. H. Sharpe , Annu. Rev. Immunol. 2016, 34, 539. 26927206 10.1146/annurev-immunol-032414-112049

[8] L. Bingle , N. J. Brown , C. E. Lewis , J. Pathol. 2002, 196, 254. 11857487 10.1002/path.1027

[9] M. E. W. Logtenberg , F. A. Scheeren , T. N. Schumacher , Immunity 2020, 52, 742. 32433947 10.1016/j.immuni.2020.04.011 PMC7340539

[10] R. Majeti , M. P. Chao , A. A. Alizadeh , W. W. Pang , S. Jaiswal , K. D. Gibbs Jr , N. van Rooijen , I. L. Weissman , Cell 2009, 138, 286. 19632179 10.1016/j.cell.2009.05.045 PMC2726837

[11] S. B. Willingham , J.‐P. Volkmer , A. J. Gentles , D. Sahoo , P. Dalerba , S. S. Mitra , J. Wang , H. Contreras‐Trujillo , R. Martin , J. D. Cohen , P. Lovelace , F. A. Scheeren , M. P. Chao , K. Weiskopf , C. Tang , A. K. Volkmer , T. J. Naik , T. A. Storm , A. R. Mosley , B. Edris , S. M. Schmid , C. K. Sun , M.‐S. Chua , O. Murillo , P. Rajendran , A. C. Cha , R. K. Chin , D. Kim , M. Adorno , T. Raveh , et al., Proc. Natl. Acad. Sci. U.S.A. 2012, 109, 6662. 22451913 10.1073/pnas.1121623109 PMC3340046

[12] J. Calderaro , B. Rousseau , G. Amaddeo , M. Mercey , C. Charpy , C. Costentin , A. Luciani , E.‐S. Zafrani , A. Laurent , D. Azoulay , F. Lafdil , J.‐M. Pawlotsky , Hepatology 2016, 64, 2038. 27359084 10.1002/hep.28710

[13] H. Yamaguchi , J.‐M. Hsu , W.‐H. Yang , M.‐C. Hung , Nat. Rev. Clin. Oncol. 2022, 19, 287. 35132224 10.1038/s41571-022-00601-9

[14] S. C. Casey , L. Tong , Y. Li , R. Do , S. Walz , K. N. Fitzgerald , A. M. Gouw , V. Baylot , I. Gütgemann , M. Eilers , D. W. Felsher , Science 2016, 352, 227. 26966191 10.1126/science.aac9935 PMC4940030

[15] L. Shi , Y. Yang , M. Li , C. Li , Z. Zhou , G. Tang , L. Wu , Y. Yao , X. Shen , Z. Hou , H. Jia , Mol. Ther. 2022, 30, 1564. 35051616 10.1016/j.ymthe.2022.01.003 PMC9077312

[16] T. L. Young , T. Matsuda , C. L. Cepko , Curr. Biol. 2005, 15, 501. 15797018 10.1016/j.cub.2005.02.027

[17] J. Long , S. S. Badal , Z. Ye , Y. Wang , B. A. Ayanga , D. L. Galvan , N. H. Green , B. H. Chang , P. A. Overbeek , F. R. Danesh , J. Clin. Invest. 2016, 126, 4205. 27760051 10.1172/JCI87927 PMC5096930

[18] J. P. Lewandowski , G. Dumbović , A. R. Watson , T. Hwang , E. Jacobs‐Palmer , N. Chang , C. Much , K. M. Turner , C. Kirby , N. D. Rubinstein , A. F. Groff , S. C. Liapis , C. Gerhardinger , A. Bester , P. P. Pandolfi , J. G. Clohessy , H. E. Hoekstra , M. Sauvageau , J. L. Rinn , Genome Biol. 2020, 21, 237. 32894169 10.1186/s13059-020-02081-5 PMC7487648

[19] Y. H. Lin , M. H. Wu , Y. H. Huang , C. T. Yeh , M. L. Cheng , H. C. Chi , C. Y. Tsai , I. H. Chung , C. Y. Chen , K. H. Lin , Hepatology 2018, 67, 188. 28802060 10.1002/hep.29462

[20] K. Katsushima , A. Natsume , F. Ohka , K. Shinjo , A. Hatanaka , N. Ichimura , S. Sato , S. Takahashi , H. Kimura , Y. Totoki , T. Shibata , M. Naito , H. J. Kim , K. Miyata , K. Kataoka , Y. Kondo , Nat. Commun. 2016, 7, 13616. 27922002 10.1038/ncomms13616 PMC5150648

[21] G. J. Goodall , V. O. Wickramasinghe , Nat. Rev. Cancer 2021, 21, 22. 33082563 10.1038/s41568-020-00306-0

[22] G. Dumbović , U. Braunschweig , H. K. Langner , M. Smallegan , J. Biayna , E. P. Hass , K. Jastrzebska , B. Blencowe , T. R. Cech , M. H. Caruthers , J. L. Rinn , Nat. Commun. 2021, 12, 3308. 34083519 10.1038/s41467-021-23221-w PMC8175569

[23] A. M. Khalil , M. Guttman , M. Huarte , M. Garber , A. Raj , D. Rivea Morales , K. Thomas , A. Presser , B. E. Bernstein , A. van Oudenaarden , A. Regev , E. S. Lander , J. L. Rinn , Proc. Natl. Acad. Sci. USA 2009, 106, 11667. 19571010 10.1073/pnas.0904715106 PMC2704857

[24] L. Yang , C. Lin , W. Liu , J. Zhang , K. A. Ohgi , J. D. Grinstein , P. C. Dorrestein , M. G. Rosenfeld , Cell 2011, 147, 773. 22078878 10.1016/j.cell.2011.08.054 PMC3297197

[25] C. Chu , K. Qu , F. L. Zhong , S. E. Artandi , H. Y. Chang , Mol. Cell 2011, 44, 667. 21963238 10.1016/j.molcel.2011.08.027 PMC3249421

[26] J. Ma , T. Chen , S. Wu , C. Yang , M. Bai , K. Shu , K. Li , G. Zhang , Z. Jin , F. He , H. Hermjakob , Y. Zhu , Nucleic Acids Res. 2019, 47, D1211. 30252093 10.1093/nar/gky869 PMC6323926

[27] T. Chen , J. Ma , Y. Liu , Z. Chen , N. Xiao , Y. Lu , Y. Fu , C. Yang , M. Li , S. Wu , X. Wang , D. Li , F. He , H. Hermjakob , Y. Zhu , Nucleic Acids Res. 2022, 50, D1522. 34871441 10.1093/nar/gkab1081 PMC8728291

[28] Y. Zhang , Y.‐X. Huang , D.‐L. Wang , B. Yang , H.‐Y. Yan , L.‐H. Lin , Y. Li , J. Chen , L.‐M. Xie , Y.‐S. Huang , J.‐Y. Liao , K.‐S. Hu , J.‐H. He , P. E. Saw , X. Xu , D. Yin , Theranostics 2020, 10, 10823. 32929382 10.7150/thno.47830 PMC7482804

[29] S. van Heesch , F. Witte , V. Schneider‐Lunitz , J. F. Schulz , E. Adami , A. B. Faber , M. Kirchner , H. Maatz , S. Blachut , C.‐L. Sandmann , M. Kanda , C. L. Worth , S. Schafer , L. Calviello , R. Merriott , G. Patone , O. Hummel , E. Wyler , B. Obermayer , M. B. Mücke , E. L. Lindberg , F. Trnka , S. Memczak , M. Schilling , L. E. Felkin , P. J. R. Barton , N. M. Quaife , K. Vanezis , S. Diecke , M. Mukai , et al., Cell 2019, 178, 242.31155234

[30] S. Rebouissou , J.‐C. Nault , J. Hepatol. 2020, 72, 215. 31954487 10.1016/j.jhep.2019.08.017

[31] A. B. El‐Khoueiry , B. Sangro , T. Yau , T. S. Crocenzi , M. Kudo , C. Hsu , T.‐Y. Kim , S.‐P. Choo , J. Trojan , T. H. R. Welling , T. Meyer , Y.‐K. Kang , W. Yeo , A. Chopra , J. Anderson , C. Dela Cruz , L. Lang , J. Neely , H. Tang , H. B. Dastani , I. Melero , Lancet 2017, 389, 2492. 28434648 10.1016/S0140-6736(17)31046-2 PMC7539326

[32] J. Liu , L. Wang , F. Zhao , S. Tseng , C. Narayanan , L. Shura , S. Willingham , M. Howard , S. Prohaska , J. Volkmer , M. Chao , I. L. Weissman , R. Majeti , PLoS One 2015, 10, e0137345. 26390038 10.1371/journal.pone.0137345 PMC4577081

[33] F. Martins , L. Sofiya , G. P. Sykiotis , F. Lamine , M. Maillard , M. Fraga , K. Shabafrouz , C. Ribi , A. Cairoli , Y. Guex‐Crosier , T. Kuntzer , O. Michielin , S. Peters , G. Coukos , F. Spertini , J. A. Thompson , M. Obeid , Nat. Rev. Clin. Oncol. 2019, 16, 563. 31092901 10.1038/s41571-019-0218-0

[34] A. V. R. Kornepati , R. K. Vadlamudi , T. J. Curiel , Nat. Rev. Cancer 2022, 22, 174. 35031777 10.1038/s41568-021-00431-4 PMC9989967

[35] J. Lo , E. Y. T. Lau , F. T. Y. So , P. Lu , V. S. F. Chan , V. C. H. Cheung , R. H. H. Ching , B. Y. L. Cheng , M. K. F. Ma , I. O. L. Ng , T. K. W. Lee , Liver Int. 2016, 36, 737. 26351778 10.1111/liv.12963

[36] B. I. Sikic , N. Lakhani , A. Patnaik , S. A. Shah , S. R. Chandana , D. Rasco , A. D. Colevas , T. O'Rourke , S. Narayanan , K. Papadopoulos , G. A. Fisher , V. Villalobos , S. S. Prohaska , M. Howard , M. Beeram , M. P. Chao , B. Agoram , J. Y. Chen , J. Huang , M. Axt , J. Liu , J.‐P. Volkmer , R. Majeti , I. L. Weissman , C. H. Takimoto , D. Supan , H. A. Wakelee , R. Aoki , M. D. Pegram , S. K. Padda , J. Clin. Oncol. 2019, 37, 946. 30811285 10.1200/JCO.18.02018 PMC7186585

[37] X. Liu , L. Liu , Z. Ren , K. Yang , H. Xu , Y. Luan , K. Fu , J. Guo , H. Peng , M. Zhu , Y.‐X. Fu , Cell Rep. 2018, 24, 2101. 30134171 10.1016/j.celrep.2018.07.062

[38] M. Klingenberg , A. Matsuda , S. Diederichs , T. Patel , J. Hepatol. 2017, 67, 603. 28438689 10.1016/j.jhep.2017.04.009

[39] D. Dominissini , S. Moshitch‐Moshkovitz , S. Schwartz , M. Salmon‐Divon , L. Ungar , S. Osenberg , K. Cesarkas , J. Jacob‐Hirsch , N. Amariglio , M. Kupiec , R. Sorek , G. Rechavi , Nature 2012, 485, 201. 22575960 10.1038/nature11112

[40] X. Zuo , Z. Chen , W. Gao , Y. Zhang , J. Wang , J. Wang , M. Cao , J. Cai , J. Wu , X. Wang , J. Hematol. Oncol. 2020, 13, 5. 31915027 10.1186/s13045-019-0839-x PMC6951025

[41] B. Sangro , P. Sarobe , S. Hervás‐Stubbs , I. Melero , Nat. Rev. Gastroenterol. Hepatol. 2021, 18, 525. 33850328 10.1038/s41575-021-00438-0 PMC8042636

[42] M. Hashimoto , A. O. Kamphorst , S. J. Im , H. T. Kissick , R. N. Pillai , S. S. Ramalingam , K. Araki , R. Ahmed , Annu. Rev. Med. 2018, 69, 301. 29414259 10.1146/annurev-med-012017-043208

[43] D. S. Thommen , T. N. Schumacher , Cancer Cell 2018, 33, 547. 29634943 10.1016/j.ccell.2018.03.012 PMC7116508

[44] M. Sznol , L. Chen , Clin. Cancer Res. 2013, 19, 1021. 23460533 10.1158/1078-0432.CCR-12-2063 PMC3702373

[45] A. Mantovani , S. Sozzani , M. Locati , P. Allavena , A. Sica , Trends Immunol. 2002, 23, 549. 12401408 10.1016/s1471-4906(02)02302-5

[46] M. A.‐O. Zhang , G. Hutter , S. A. Kahn , T. D. Azad , S. Gholamin , C. Y. Xu , J. Liu , A. S. Achrol , C. Richard , P. Sommerkamp , M. K. Schoen , M. N. McCracken , R. Majeti , I. Weissman , S. S. Mitra , S. H. Cheshier , PLoS One 2016, 11, e0153550. 27092773 10.1371/journal.pone.0153550 PMC4836698

[47] C. Xu , C. M. Fillmore , S. Koyama , H. Wu , Y. Zhao , Z. Chen , G. S. Herter‐Sprie , E. A. Akbay , J. H. Tchaicha , A. Altabef , J. B. Reibel , Z. Walton , H. Ji , H. Watanabe , P. A. Jänne , D. H. Castrillon , A. K. Rustgi , A. J. Bass , G. J. Freeman , R. F. Padera , G. Dranoff , P. S. Hammerman , C. F. Kim , K.‐K. Wong , Cancer Cell 2014, 25, 590. 24794706 10.1016/j.ccr.2014.03.033 PMC4112370

[48] Q. Xi , J. Zhang , G. Yang , L. Zhang , Y. Chen , C. Wang , Z. Zhang , X. Guo , J. Zhao , Z. Xue , Y. Li , Q. Zhang , Y. Da , L. Liu , Z. Yao , R. Zhang , J. Immunother Cancer 2020, 8, e000253. 32503944 10.1136/jitc-2019-000253 PMC7279671

[49] X. Zhu , B. Lan , X. Yi , C. He , L. Dang , X. Zhou , Y. Lu , Y. Sun , Z. Liu , X. Bai , K. Zhang , B. Li , M. J. Li , Y. Chen , L. Zhang , Nucleic Acids Res. 2020, 48, 6563. 32459350 10.1093/nar/gkaa441 PMC7337902

[50] A. Lánczky , B. Győrffy , J. Med. Internet Res. 2021, 23, e27633. 34309564 10.2196/27633 PMC8367126

[51] T. Li , J. Fu , Z. Zeng , D. Cohen , J. Li , Q. Chen , B. Li , X. S. Liu , Nucleic Acids Res. 2020, 48, W509. 32442275 10.1093/nar/gkaa407 PMC7319575

[52] J.‐H. Li , S. Liu , H. Zhou , L.‐H. Qu , J.‐H. Yang , Nucleic Acids Res. 2014, 42, D92. 24297251 10.1093/nar/gkt1248 PMC3964941

[53] D. Karagkouni , M. D. Paraskevopoulou , S. Tastsoglou , G. Skoufos , A. Karavangeli , V. Pierros , E. Zacharopoulou , A. G. Hatzigeorgiou , Nucleic Acids Res. 2020, 48, D101. 31732741 10.1093/nar/gkz1036 PMC7145509

[54] U. K. Muppirala , V. G. Honavar , D. Dobbs , BMC Bioinformatics 2011, 12, 489. 22192482 10.1186/1471-2105-12-489 PMC3322362

[55] F. Agostini , A. Zanzoni , P. Klus , D. Marchese , D. Cirillo , G. G. Tartaglia , Bioinformatics 2013, 29, 2928. 23975767 10.1093/bioinformatics/btt495 PMC3810848

[56] H. M. Berman , J. Westbrook , Z. Feng , G. Gilliland , T. N. Bhat , H. Weissig , I. N. Shindyalov , P. E. Bourne , Nucleic Acids Res. 2000, 28, 235. 10592235 10.1093/nar/28.1.235 PMC102472

[57] Y. Zhang , J. Wang , Y. Xiao , J. Mol. Biol. 2022, 434, 167452. 35662453 10.1016/j.jmb.2022.167452

