# Supplementary material for: M6A‐mediated upregulation of lncRNA TUG1 in liver cancer cells regulates the antitumor response of CD8+ T cells and phagocytosis of macrophages
Source: Adv Sci (Weinh). 2024 Jul 9;11(34):2400695. doi: 10.1002/advs.202400695 (PMC11425850; doi:10.1002/advs.202400695)
Supplement: Supplementary file 1 — Supporting Information [file ADVS-11-2400695-s001.docx]

Supporting Information

**M^6^A-mediated upregulation of lncRNA TUG1 in liver cancer cells regulates the antitumor response of CD8^+^ T cells and phagocytosis of macrophages**

*Qing Xi*, Guangze Yang, Xue He,* *Hao Zhuang, Li Li, Bing Lin, Lingling Wang, Xianyang Wang, Chunqiang Fang, Qiurui Chen, Yongjie Yang, Zhaoan Yu, Hao Zhang, Wenqian Cai, Yan Li, Han Shen, Li Liu, Rongxin Zhang**

**Figure S1**


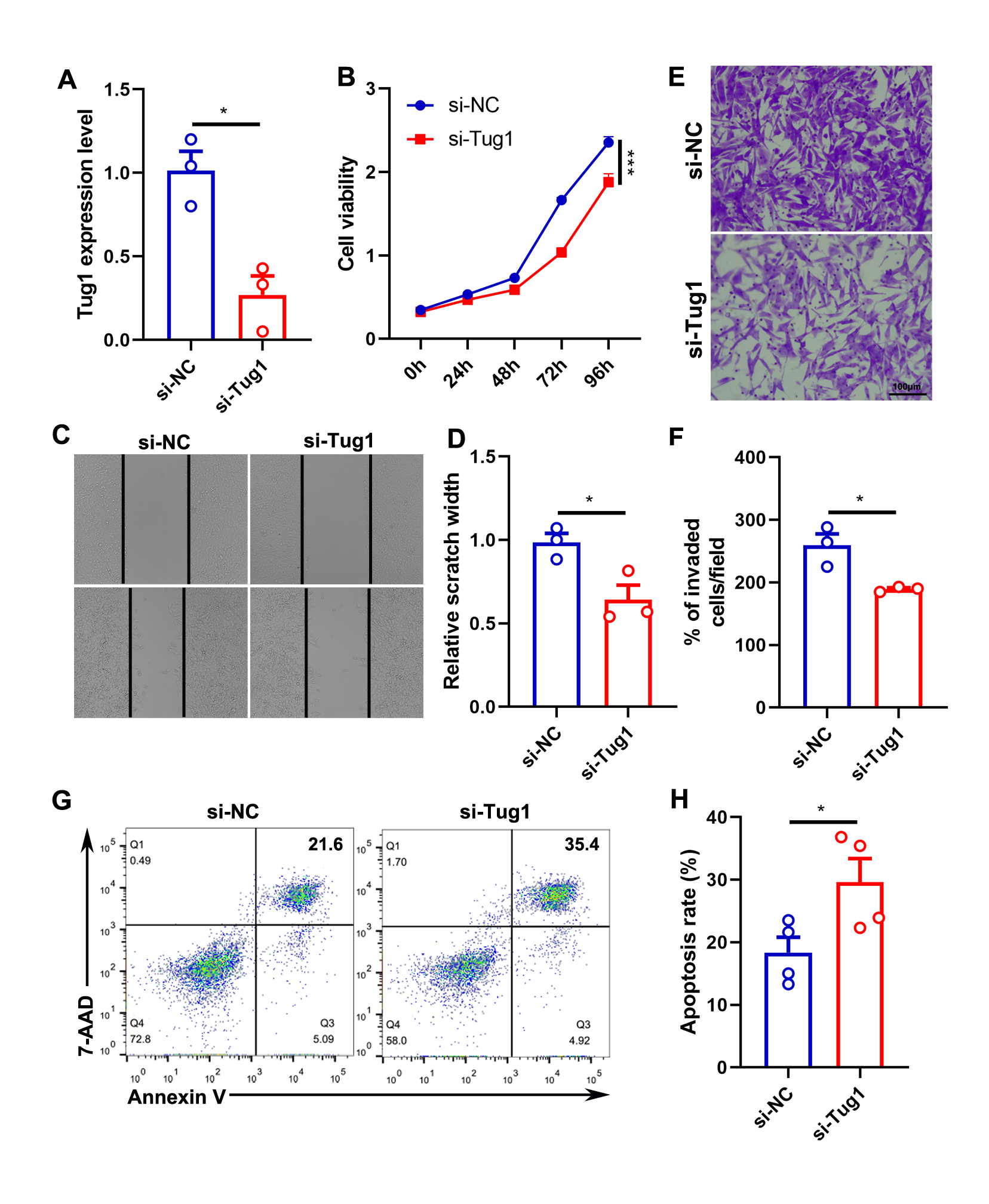


**Figure S1. Knockdown of Tug1 inhibits proliferation, migration and promotes apoptosis of HCC cells. (A)** The qRT-PCR was performed to determine the expression of Tug1 in the control (si-NC) and Tug1 siRNAs (si-Tug1) transfected Hepa1-6 cells. **(B)** The cell counting kit-8 (CCK8) assay was performed to assess cell proliferation in si-NC and si-Tug1 transfected Hepa1-6 cells. **(C-D)** The wound healing assay was performed in si-NC and si-Tug1 transfected Hepa1-6 cells (n = 3). Representative images and quantitative analysis were shown. **(E-F)** The transwell invasion assay was performed in si-NC and si-Tug1 transfected Hepa1-6 cells (n = 3). Representative images and quantitative analysis were shown. **(G-H)** The cell apoptosis assay was performed in si-NC and si-Tug1 transfected Hepa1-6 cells and analyzed by flow cytometry (n = 4). Representative images and quantitative analysis were shown. Results are represented as the mean ± SEM. Comparisons between groups were determined by unpaired two-tailed Student’s t-test. *, p<0.05; ***, p<0.001.

**Figure S2**


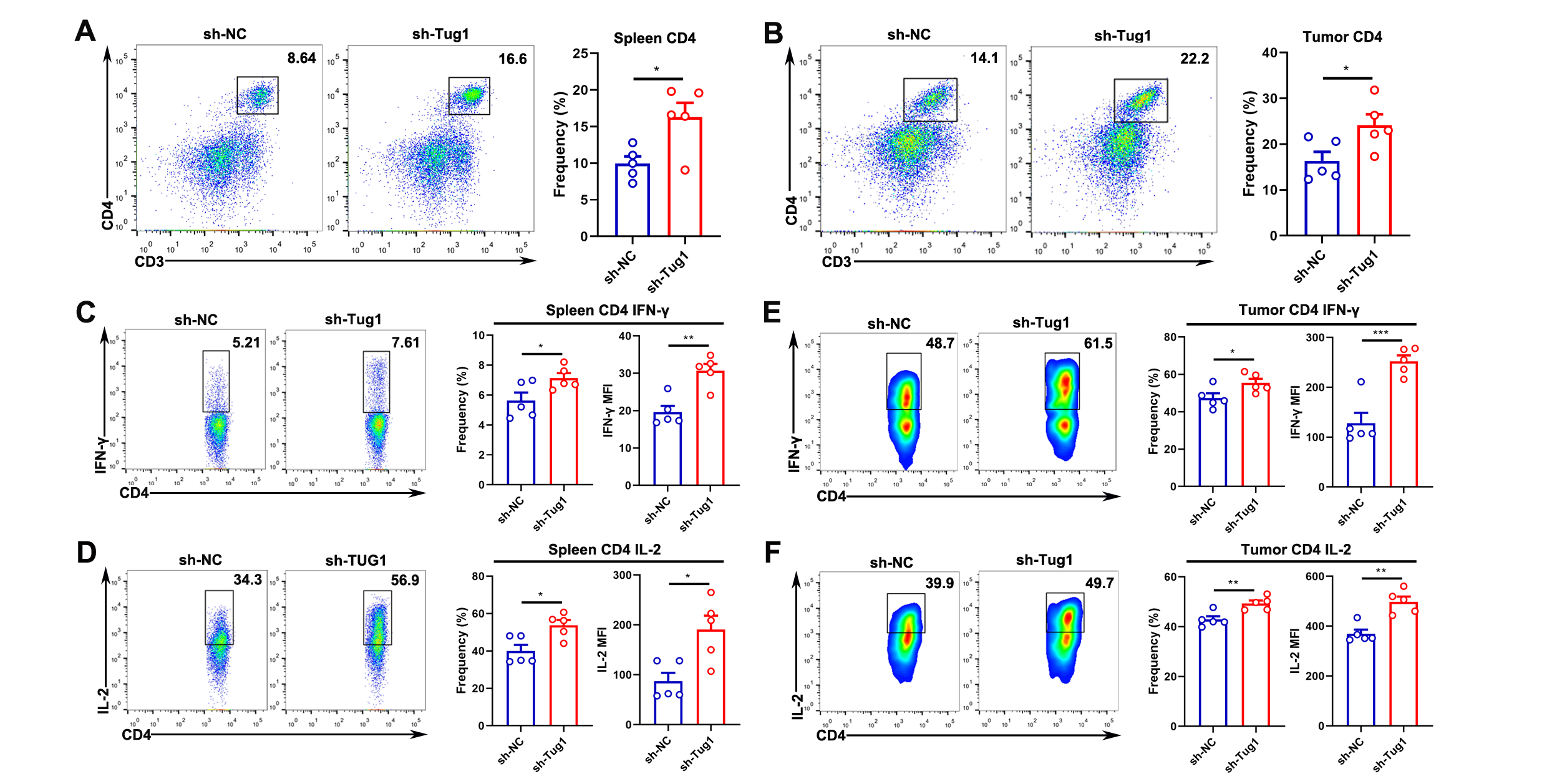


**Figure S2. Knockdown the expression of Tug1 notably promote the anti-tumor function of CD4^+^ T cells. (A)** The frequencies of CD4^+^ T cells in the spleens from mice bearing sh-NC and sh-Tug1 Hepa1-6 cells (n = 5). **(B)** The frequencies of CD4^+^ T cells in the tumors from mice bearing sh-NC and sh-Tug1 Hepa1-6 cells (n = 5). **(C-D)** The secretion of cytokines of CD4^+^ T cells in the spleens from mice bearing sh-NC and sh-Tug1 Hepa1-6 cells (n = 5). **(E-F)** The secretion of cytokines of CD4^+^ T cells in the tumors from mice bearing sh-NC and sh-Tug1 Hepa1-6 cells (n = 5). The error bars were shown as mean ± SEM. Comparisons between groups were determined by the unpaired two-tailed Student’s t-test. *, p<0.05; **, p<0.01; ***, p<0.001.

**Figure S3**


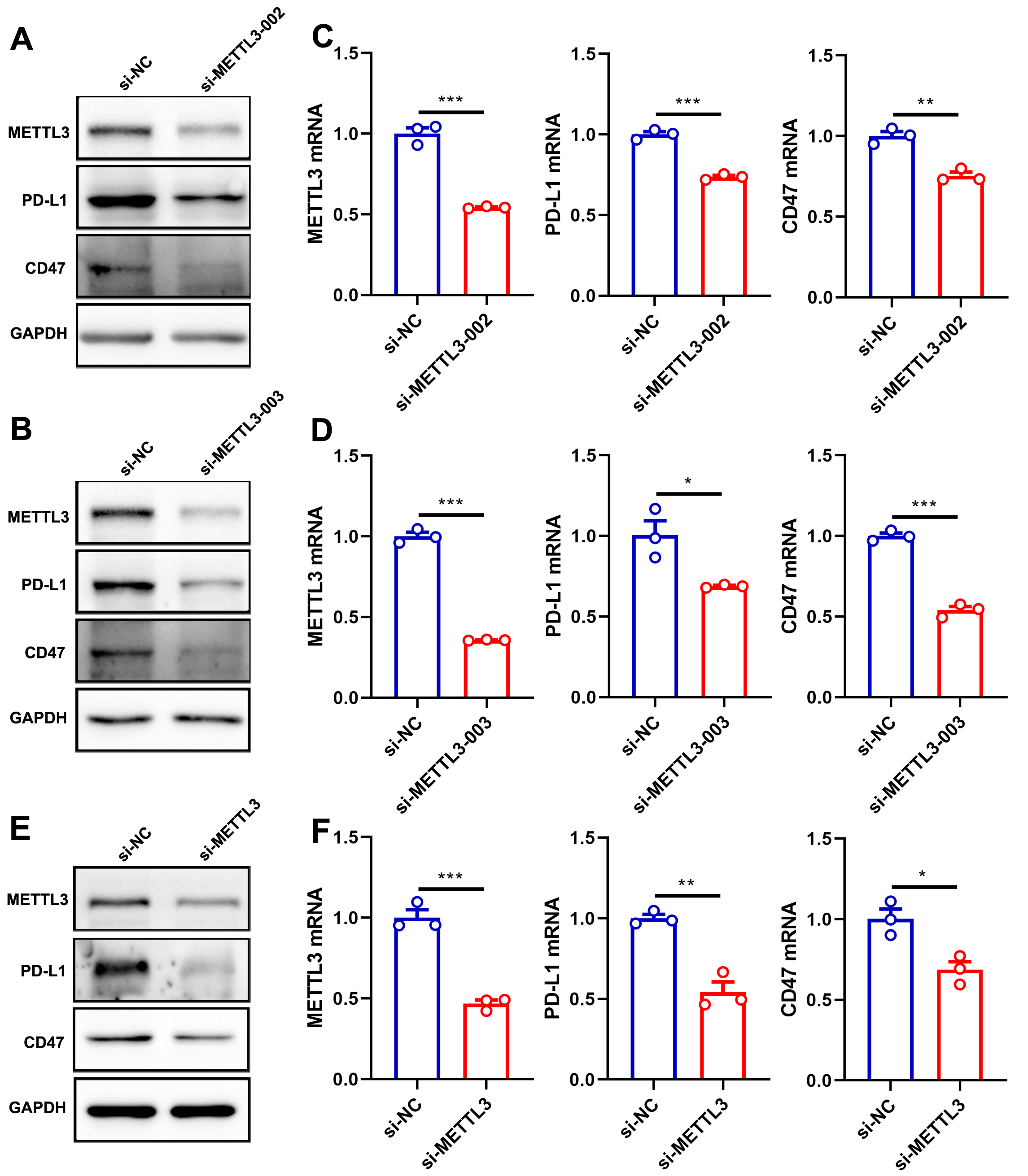


**Figure S3. Downregulation of METTL3 significantly decreased PD-L1 and CD47 expression. (A)** The expression of METTL3, PD-L1 and CD47 in protein levels with si-NC and si-METTL3-002 transfected HepG2 cells. **(B)** The expression of METTL3, PD-L1 and CD47 in protein levels with si-NC and si-METTL3-003 transfected HepG2 cells. (**C**) The expression of METTL3, PD-L1 and CD47 in mRNA levels with si-NC and si-METTL3-002 transfected HepG2 cells. (**D**) The expression of METTL3, PD-L1 and CD47 in mRNA levels with si-NC and si-METTL3-003 transfected HepG2 cells. **(E)** The expression of METTL3, PD-L1 and CD47 in protein levels with si-NC and si-METTL3-001 transfected LM3 cells. (**F**) The expression of METTL3, PD-L1 and CD47 in mRNA levels with si-NC and si-METTL3-001 transfected LM3 cells.The error bars were shown as mean ± SEM. Statistical analysis was performed using the unpaired two-tailed Student’s t-test. *, p<0.05;**, p<0.01; ***, p<0.001.

**Figure S4**


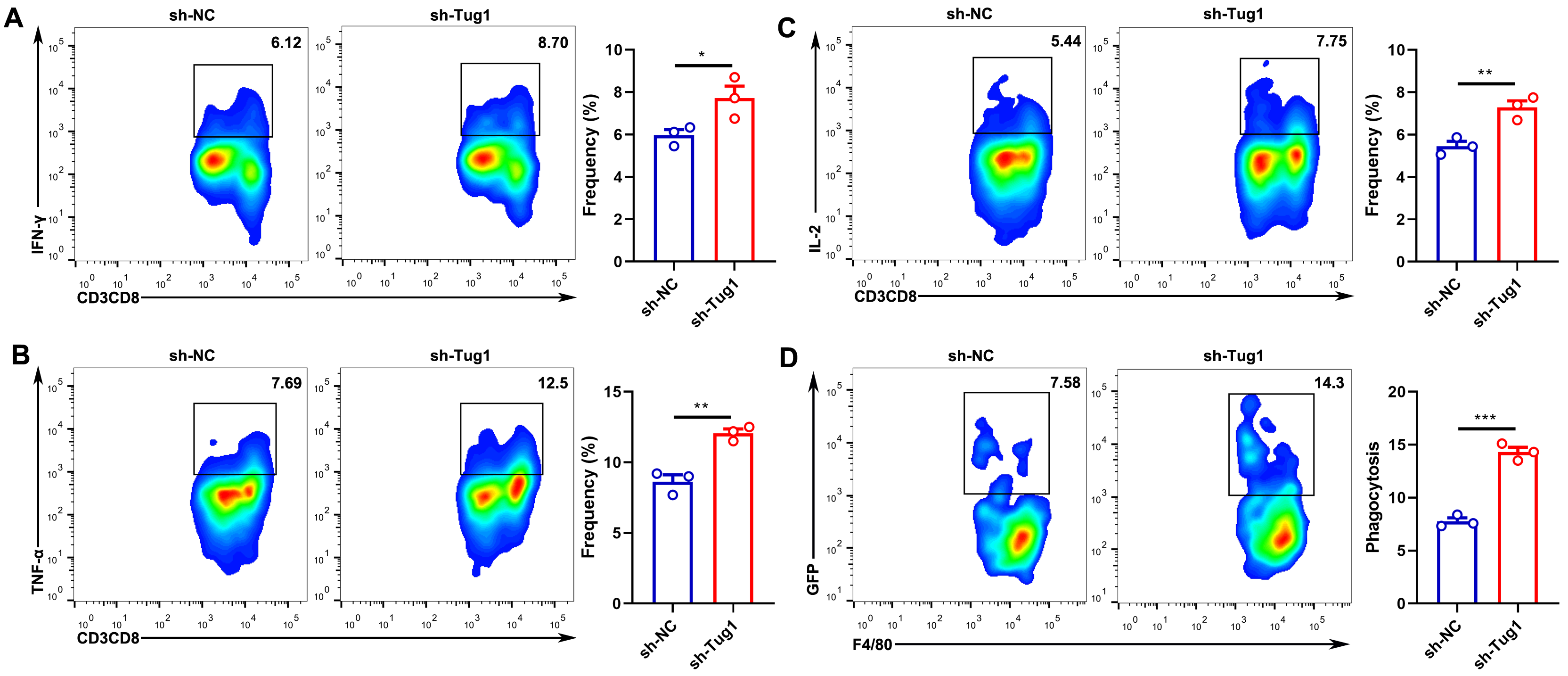


**Figure S4.** **Knockdown of Tug1 in Hepa1c1c7 cells could restore the activation of CD8^+^ T cells, and enhance the phagocytosis to cancer cells by macrophages.** **(A-C)** The isolated CD8^+^ T cells were co-cultured with sh-NC or sh-Tug1 Hepa1c1c7 cells. the secretion of IFN-γ, TNF-α and IL-2 were increased in CD8^+^ T cells co-cultured with sh-Tug1 Hepa1c1c7 cells (n = 3). **(D)** The peritoneal cavity-derived macrophages were co-cultured with sh-NC or sh-Tug1 Hepa1c1c7 cells, the phagocytosis of sh-Tug1 Hepa1c1c7 cells by peritoneal cavity-derived macrophages increased significantly detected by flow cytometry (n = 3). Results are represented as the mean ± SEM. Statistical analysis was performed using the unpaired two-tailed Student’s t-test. *, p<0.05;**, p<0.01; ***, p<0.001.

**Figure S5**


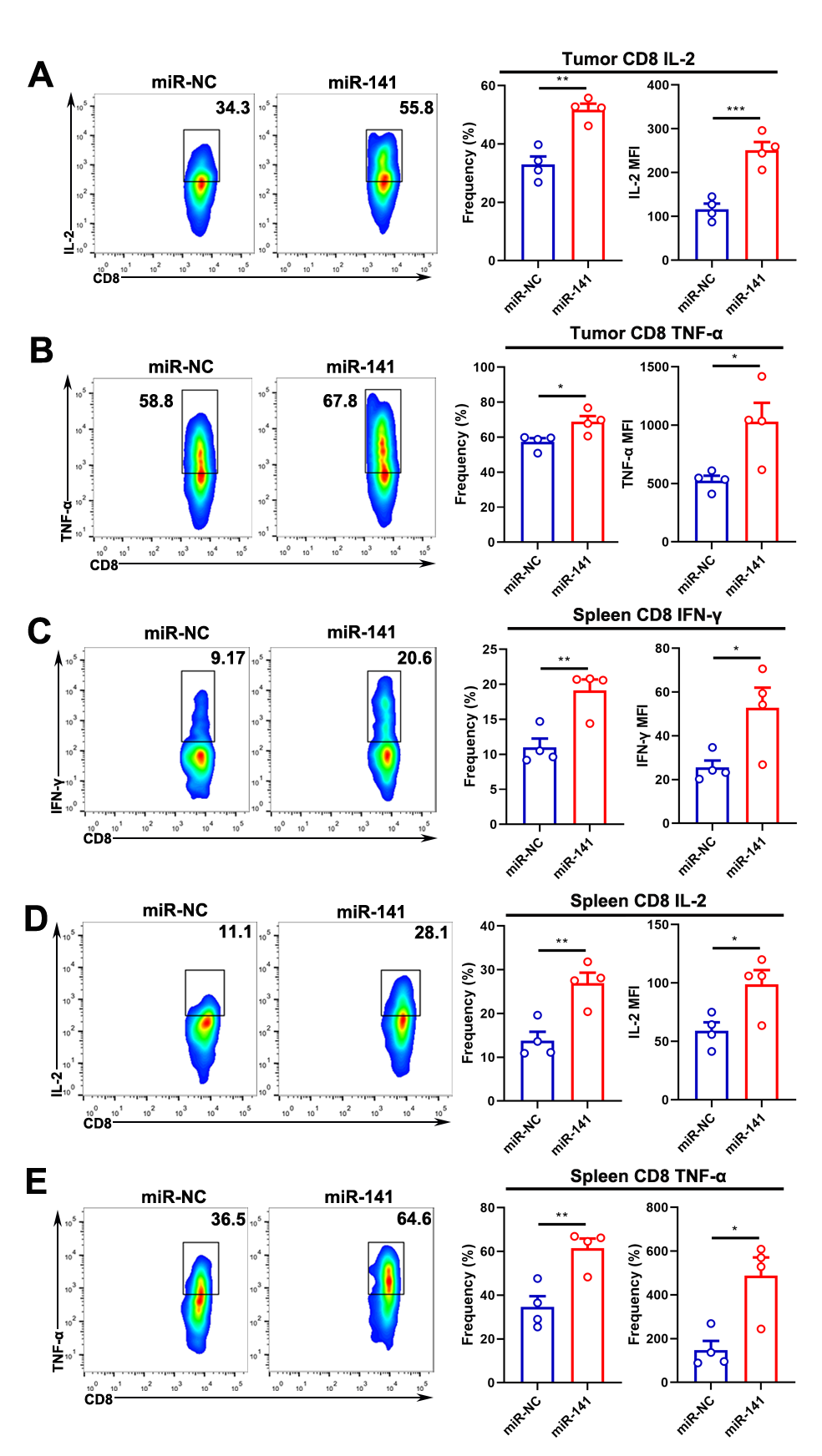


**Figure S5.** **Overexpression of miR-141 enhances antitumor immunity.** The miR-NC and miR-141 overexpression Hepa1-6 cells were injected subcutaneously into mice and euthanized at indicated time after tumor transplantation. **(A-B)** The secretion of cytokines of CD8^+^ T cells in the tumors from mice bearing miR-NC and miR-141 overexpression Hepa1-6 cells (n = 4). **(C-E)** The secretion of cytokines of CD8^+^ T cells in the spleens from mice bearing miR-NC and miR-141 overexpression Hepa1-6 cells (n = 4). Results are represented as the mean ± SEM. Statistical analysis was performed using the unpaired two-tailed Student’s t-test. *, p<0.05; **, p<0.01; ***, p<0.001.

**Figure S6**


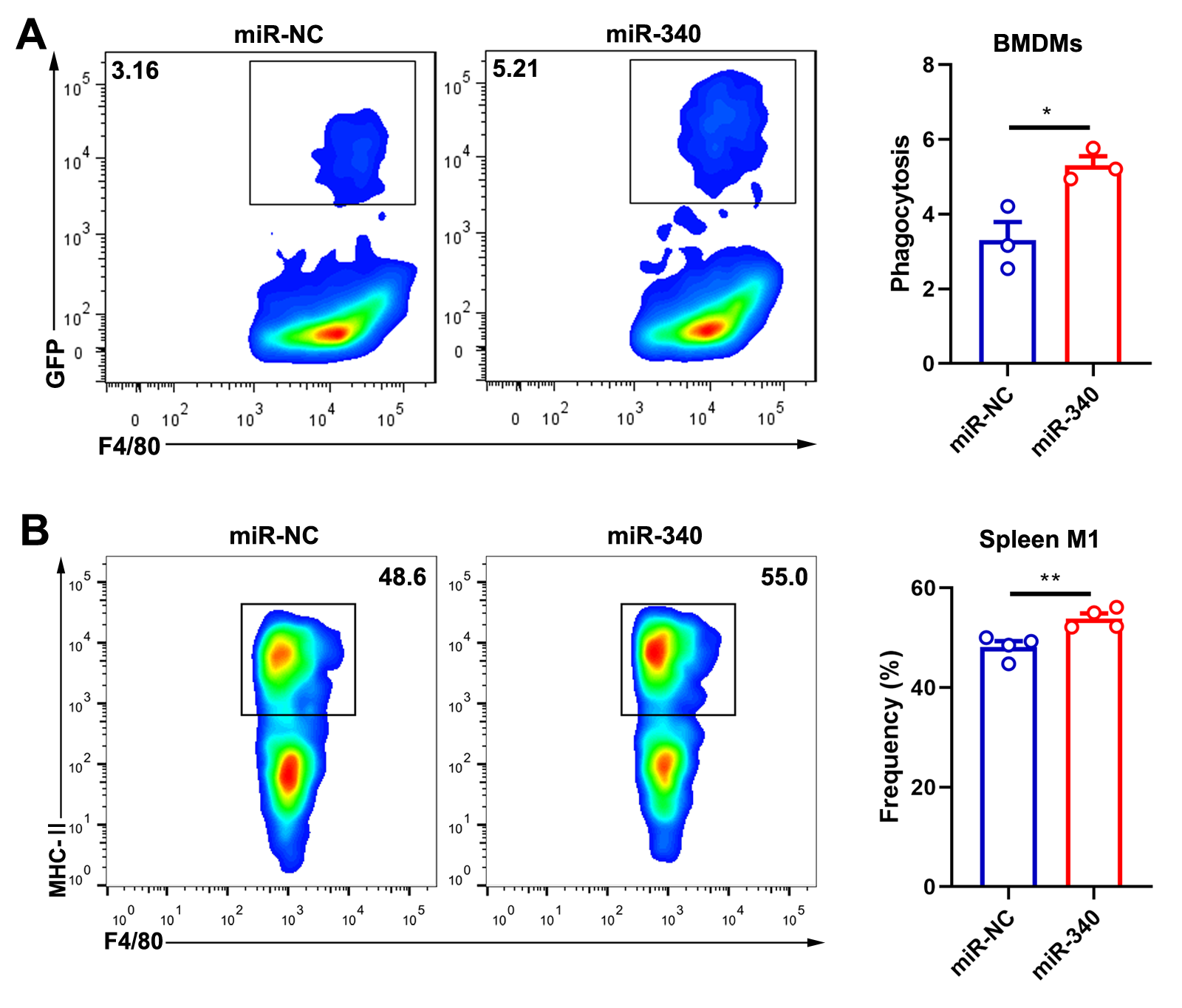


**Figure S6. Overexpression of miR-340 promoted phagocytosis of macrophages to HCC cells. (A)** Representative plots andstatistical analysis of F4/80^+^ BMDMs phagocytosing co-cultured miR-NC or miR-340 overexpression Hepa1-6 cells (n = 3). **(B)** The frequency of M1-like (CD11b^+^F4/80^+^MHC-II^+^) macrophages in the spleens from mice bearing miR-NC and miR-340 overexpression Hepa1-6 cells (n = 4). Results are represented as the mean ± SEM. Statistical analysis was performed using the unpaired two-tailed Student’s t-test. *, p<0.05; **, p<0.01.

**Figure S7**


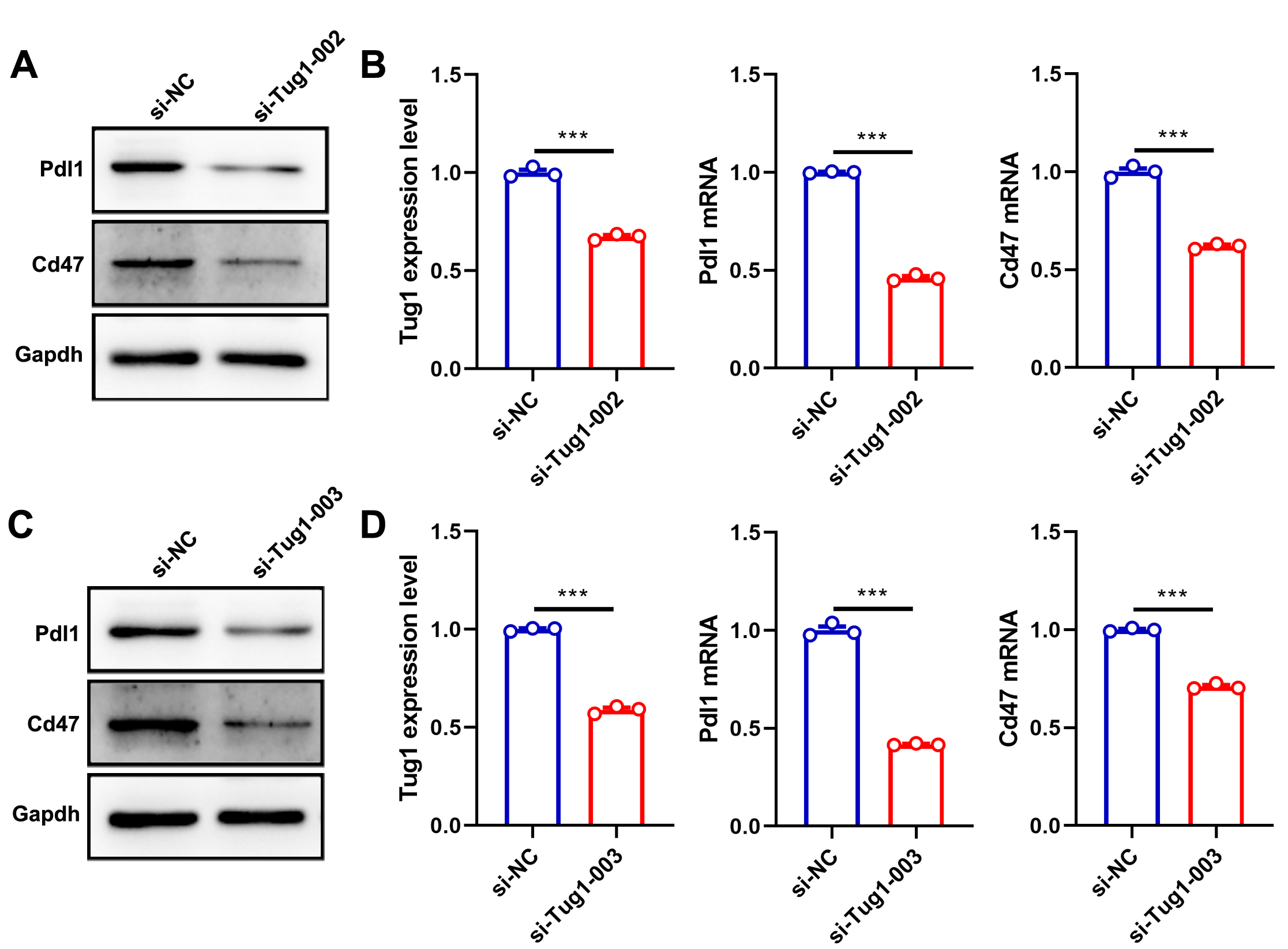


**Figure S7. Downregulation of Tug1 significantly decreased Pdl1 and Cd47 expression.** **(A)** The expression of Pdl1 and Cd47 in protein levels with si-NC and si-Tug1-002 transfected Hepa1-6 cells. **(B)** The expression of Pdl1 and Cd47 in mRNA levels with si-NC and si-Tug1-002 transfected Hepa1-6 cells. (**C**) The expression of Pdl1 and Cd47 in protein levels with si-NC and si-Tug1-003 transfected Hepa1-6 cells. (**D**) The expression of Pdl1 and Cd47 in mRNA levels with si-NC and si-Tug1-003 transfected Hepa1-6 cells. Results are represented as the mean ± SEM. Statistical analysis was performed using the unpaired two-tailed Student’s t-test. ***, p<0.001.

**Figure S8**


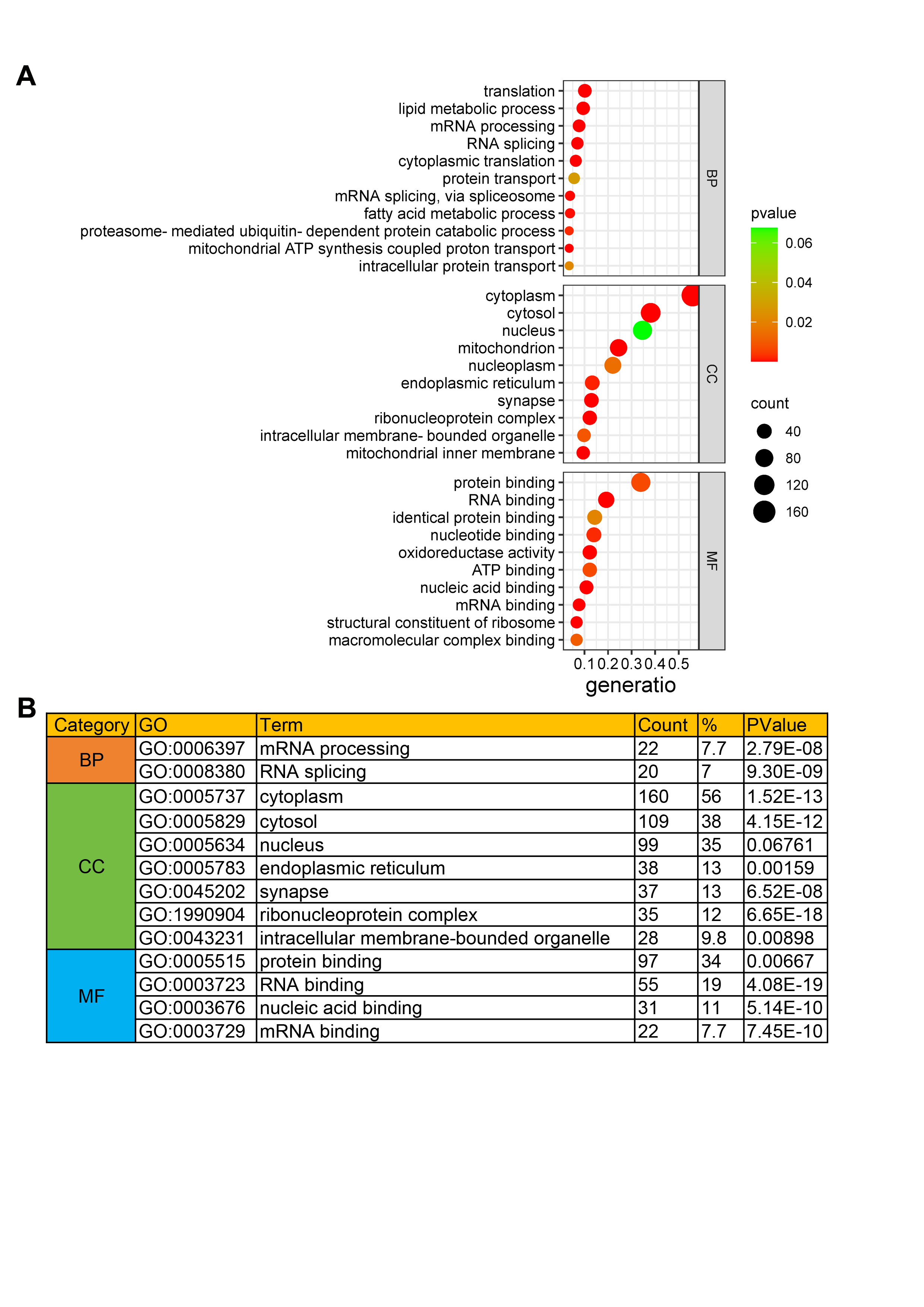


**Figure S8. The analysis of GO terms of Tug1-related proteins obtained by ChIRP-MS. (A)** GO terms of Tug1-related proteins were performed on screened genes. The terms for biological processes (BP), cellular component (CC) and molecular function (MF) are shown. **(B)** GO terms of Tug1-related proteins related to Ybx1 were shown.

**Figure S9**


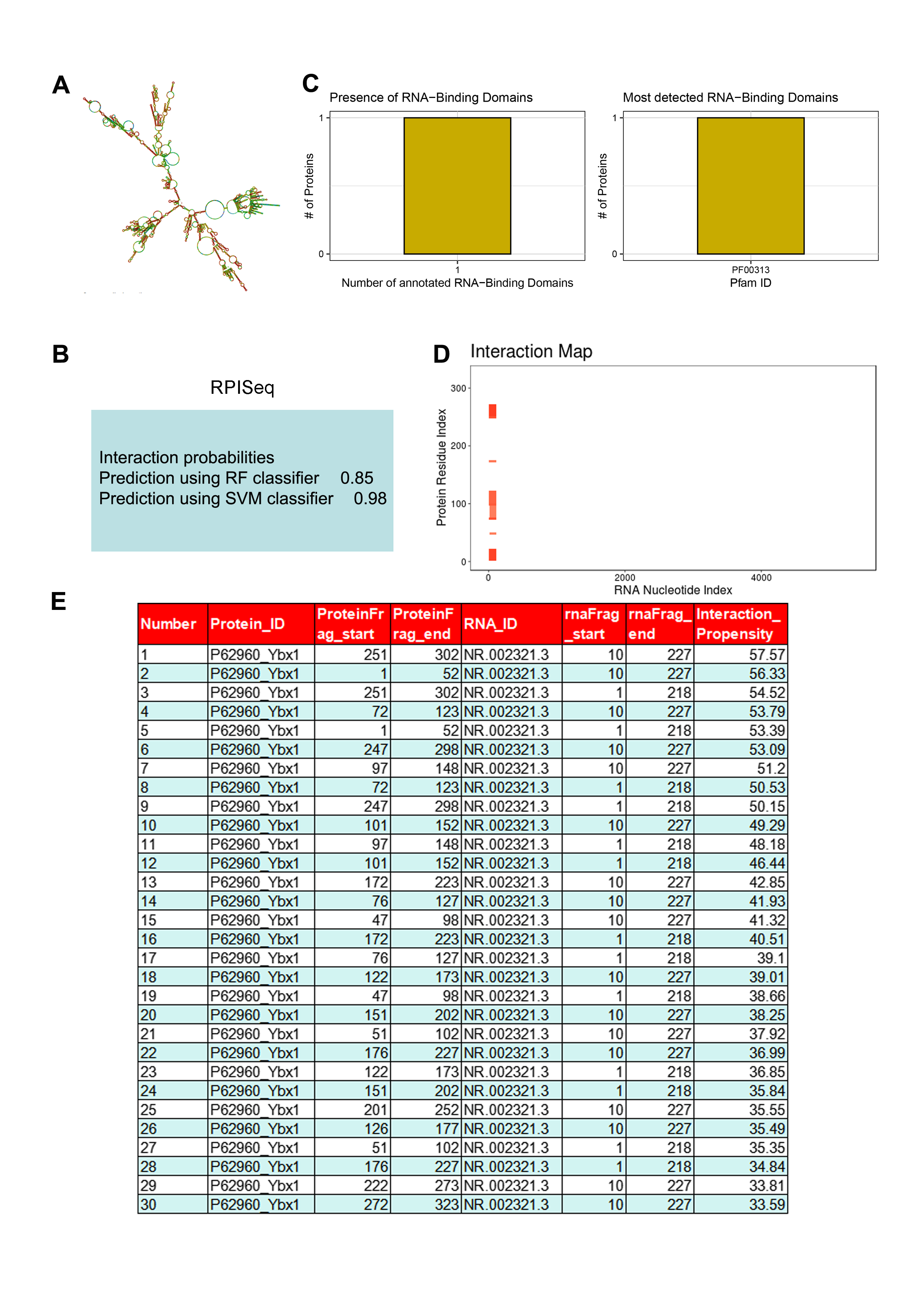


**Figure S9. Bioinformatics predicts the binding potential regions of Tug1 to Ybx1 protein. (A)** RNAfold web server predicts the RNA structure of Tug1. **(B)** RPISeq predicted the binding potential regions of Tug1 to Ybx1 protein. **(C-E)** CatRAPID predicted the top 30 binding potential regions of Tug1 to Ybx1 protein.

**Figure S10**


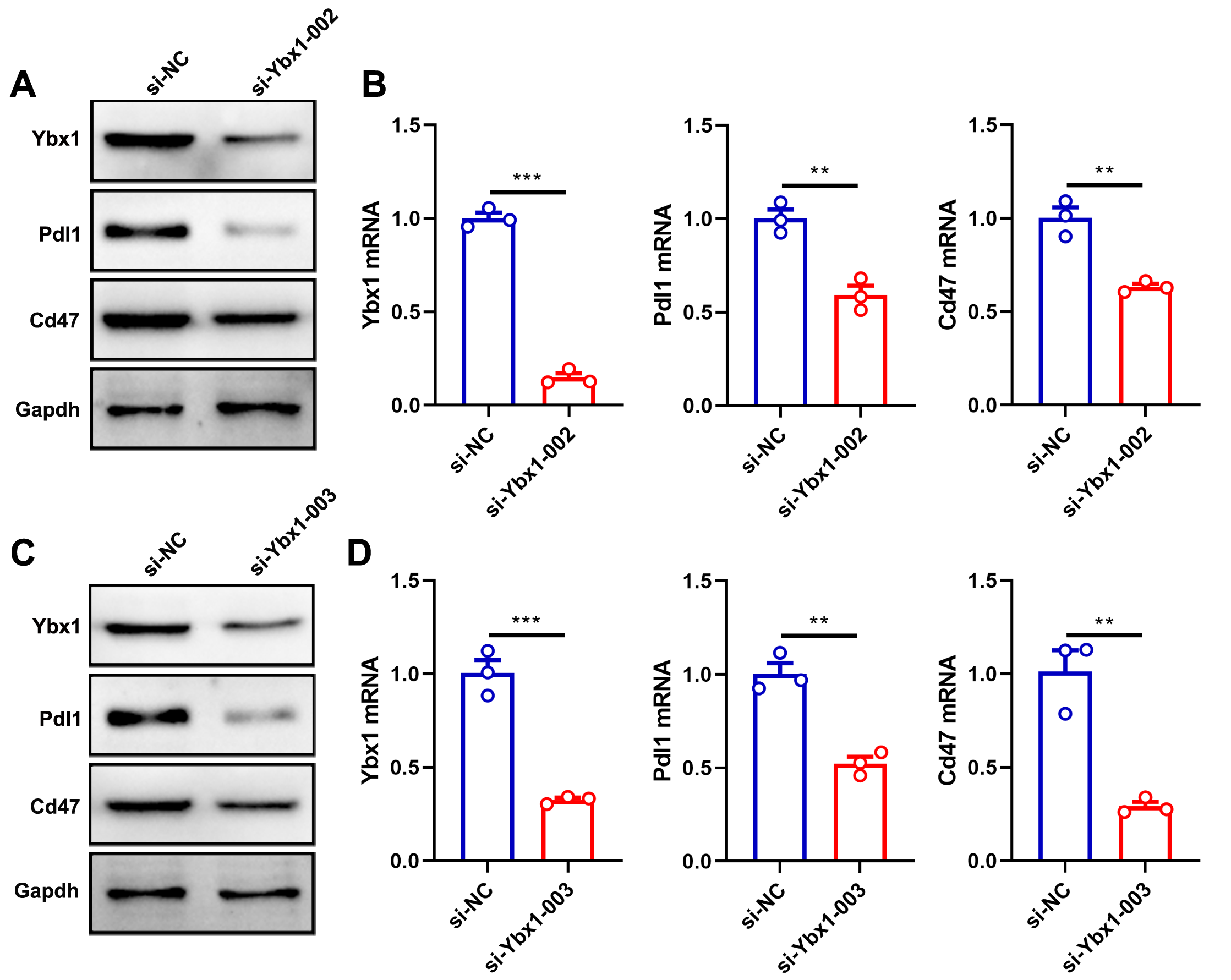


**Figure S10. Downregulation of Ybx1 significantly decreased Pdl1 and Cd47 expression. (A)** The expression of Ybx1, Pdl1 and Cd47 in protein levels with si-NC and si-Ybx1-002 transfected Hepa1-6 cells. (**B**) The expression of Ybx1, Pdl1 and Cd47 in mRNA levels with si-NC and si-Ybx1-002 transfected Hepa1-6 cells. **(C)** The expression of Ybx1, Pdl1 and Cd47 in protein levels with si-NC and si-Ybx1-003 transfected Hepa1-6 cells. (**D**) The expression of Ybx1, Pdl1 and Cd47 in mRNA levels with si-NC and si-Ybx1-003 transfected Hepa1-6 cells. Results are represented as the mean ± SEM. Statistical analysis was performed using the unpaired two-tailed Student’s t-test.**, p<0.01; ***, p<0.001.

**Figure S11**


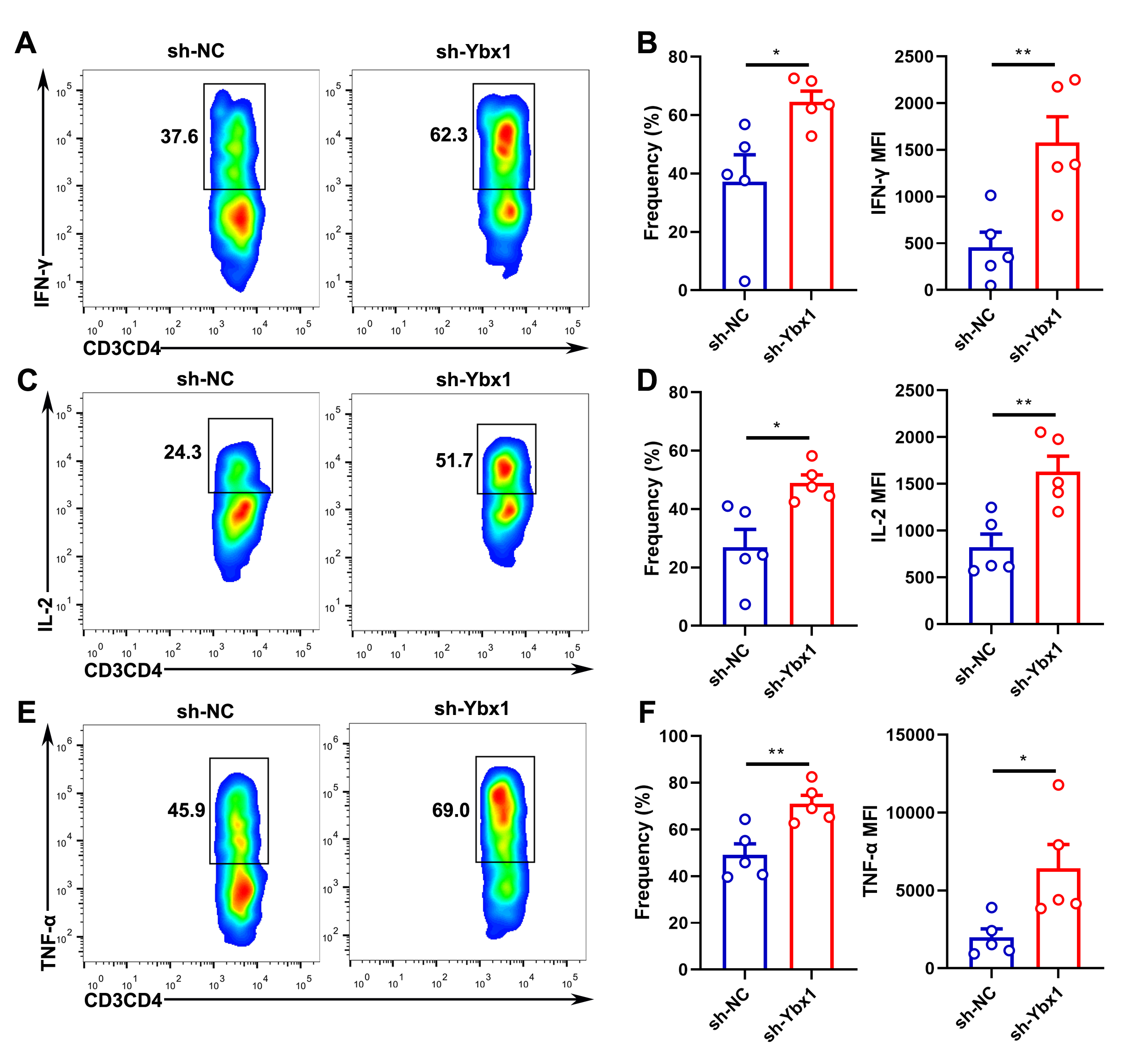


**Figure S11. Knockdown the expression of Ybx1 notably promote the anti-tumor function of CD4^+^ T cells.** The sh-NC and sh-Ybx1 Hepa1-6 cells were injected subcutaneously into mice and euthanized at indicated time after tumor transplantation. **(A)** Representative images of IFN-γ section from CD4^+^ T cells in the tumors from mice bearing sh-NC and sh-Ybx1 Hepa1-6 cells. **(B)** The frequencies and MFI of IFN-γ section from CD4^+^ T cells in the tumors from mice bearing sh-NC and sh-Ybx1 Hepa1-6 cells (n = 5). **(C)** Representative images of IL-2 section from CD4^+^ T cells in the tumors from mice bearing sh-NC and sh-Ybx1 Hepa1-6 cells. **(D)** The frequencies and MFI of IL-2 section from CD4^+^ T cells in the tumors from mice bearing sh-NC and sh-Ybx1 Hepa1-6 cells (n = 5). **(E)** Representative images of TNF-α section from CD4^+^ T cells in the tumors from mice bearing sh-NC and sh-Ybx1 Hepa1-6 cells. **(F)** The frequencies and MFI of TNF-α section from CD4^+^ T cells in the tumors from mice bearing sh-NC and sh-Ybx1 Hepa1-6 cells (n = 5). Results are represented as the mean ± SEM. Statistical analysis was performed using the unpaired two-tailed Student’s t-test. *, p<0.05; **, p<0.01.

**Figure S12**


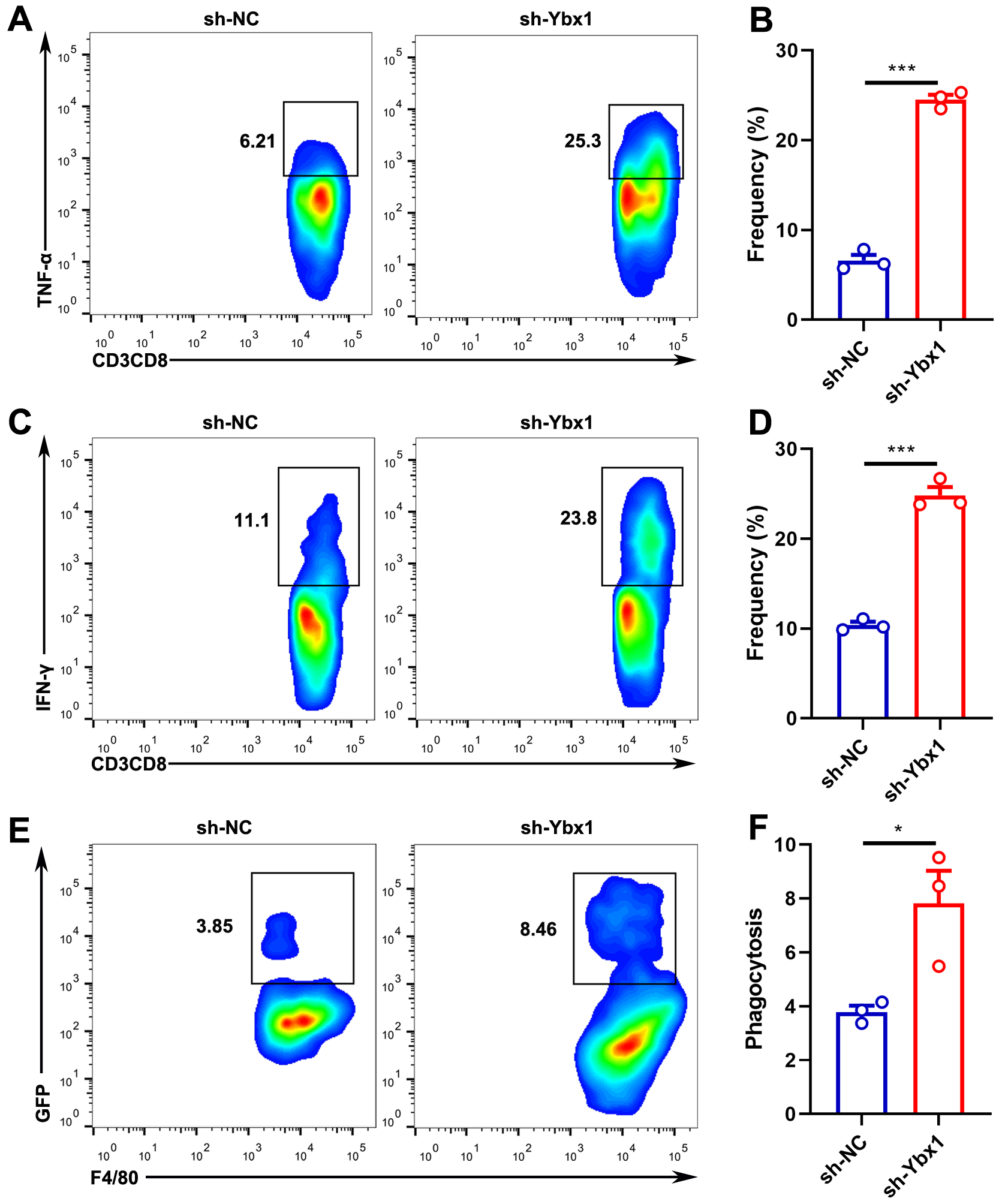


**Figure S12. Knockdown of Ybx1 in Hepa1c1c7 cells could restore the activation of CD8^+^ T cells and increased phagocytosis to cancer cells by macrophages. (A-D)** The isolated CD8^+^ T cells were co-cultured with sh-NC or sh-Ybx1 Hepa1c1c7 cells. the secretion of TNF-α and IFN-γ were increased in CD8^+^ T cells co-cultured with sh-Ybx1 Hepa1c1c7 cells (n = 3). **(E-F)** The peritoneal cavity-derived macrophages were co-cultured with sh-NC or sh-Ybx1 Hepa1c1c7 cells, the phagocytosis of sh-Ybx1 Hepa1c1c7 cells by peritoneal cavity-derived macrophages increased significantly detected by flow cytometry (n = 3). Results are represented as the mean ± SEM. Statistical analysis was performed using the unpaired two-tailed Student’s t-test. *, p<0.05;***, p<0.001.

**Figure S13**


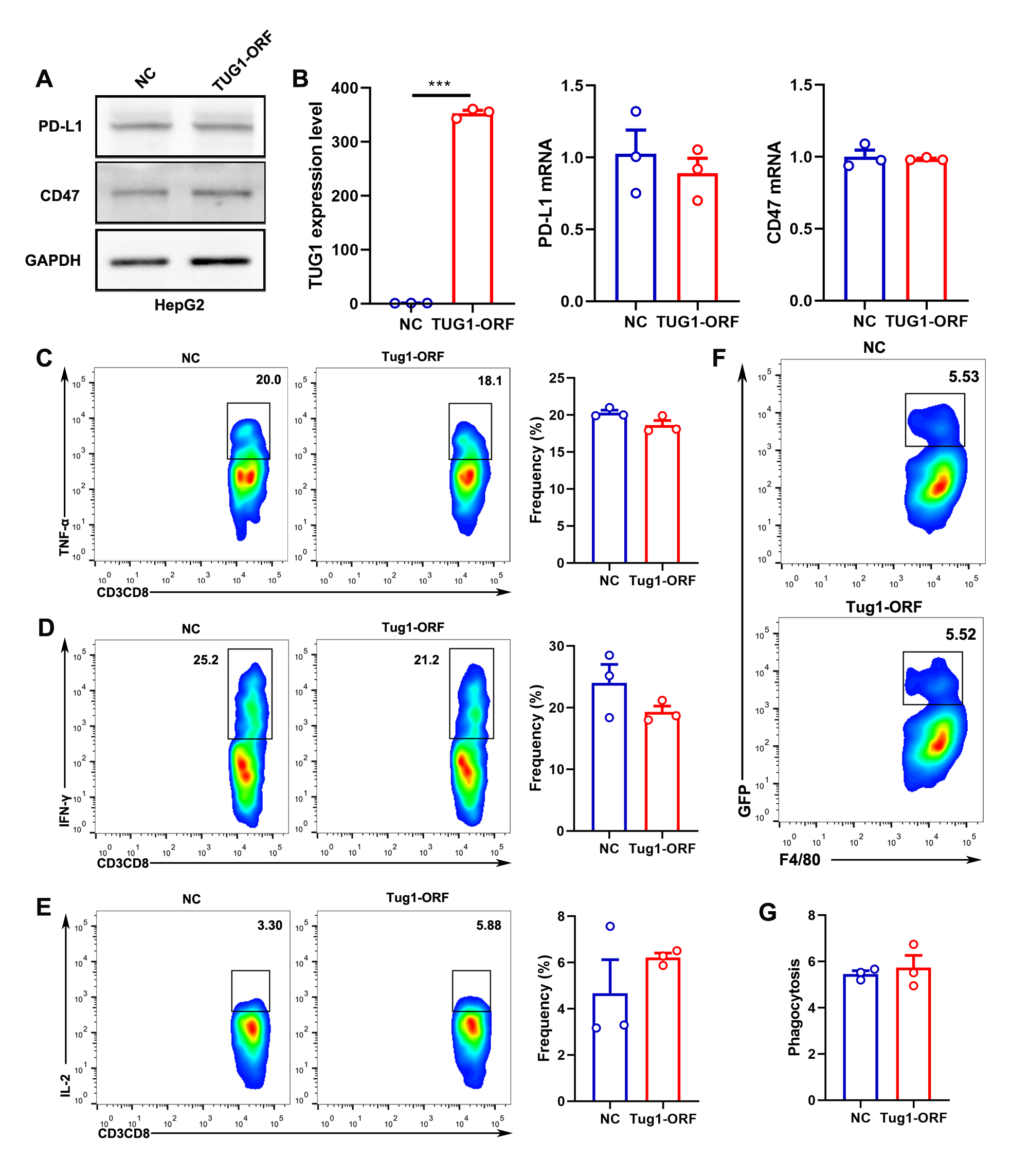


**Figure S13. TUG1-ORF did not impact PD-L1 and CD47 expression, as well as the activation of CD8^+^ T cells and phagocytosis to cancer cells by macrophages. (A)** The expression of PD-L1 and CD47 in protein levels with NC and TUG1-ORF transfected HepG2 cells. **(B)** The expression of PD-L1 and CD47 in mRNA levels with NC and TUG1-ORF transfected HepG2 cells. **(C-E)** The isolated CD8^+^ T cells were co-cultured with NC or Tug1-ORF transfected Hepa1-6 cells, the secretion of TNF-α, IFN-γ and IL-2 were detected by flow cytometry (n = 3). **(F-G)** The peritoneal cavity-derived macrophages were co-cultured with NC or Tug1-ORF transfected Hepa1-6 cells, the phagocytosis of macrophages were detected by flow cytometry (n = 3). Results are represented as the mean ± SEM. Statistical analysis was performed using the unpaired two-tailed Student’s t-test. ***, p<0.001.

**Table S1. The primers used for real-time qPCR are listed.**

| **Gene** | **Species** | **Forward primer** | **Reverse primer** |
| --- | --- | --- | --- |
| Tug1 | mouse | CAAGAAACAGCAACACCAGAAG | TAAGGTCCCCATTCAAGTCAGT |
| Cd47 | mouse | TGCGGTTCAGCTCAACTACTG | GCTTTGCGCCTCCACATTAC |
| Pdl1 | mouse | GCTCCAAAGGACTTGTACGTG | TGATCTGAAGGGCAGCATTTC |
| Gapdh | mouse | CCATGTTTGTGATGGGTGTGAACCA | ACCAGTGGATGCAGGGATGATGTTC |
| Ybx1 | mouse | CAGACCGTAACCATTATAGACGC | ATCCCTCGTTCTTTTCCCCAC |
| Mettl3 | mouse | CTGGGCACTTGGATTTAAGGAA | TGAGAGGTGGTGTAGCAACTT |
| TUG1 | human | CTCTCTTTACTGAGGGTGCTTTAGCT | TCTCTCCATATTTTGGCTCTGCTT |
| GAPDH | human | ACAACTTTGGTATCGTGGAAGG | GCCATCACGCCACAGTTTC |
| YBX1 | human | TCGCCAAAGACAGCCTAGAGA | TCTGCGTCGGTAATTGAAGTTG |
| METTL3 | human | AGCCTTCTGAACCAACAGTCC | CCGACCTCGAGAGCGAAAT |
| CD47 | human | GGCAATGACGAAGGAGGTTA | ATCCGGTGGTATGGATGAGA |
| PD-L1 | human | CCAGTCACCTCTGAACATG | TCAGTGTGCTGGTCACATTG |

**Table S2. The antibodies used in this study are listed.**

| **Antibodies** | **Supplier** | **Application** | **Catalog number** | **CloneNo.** | **Dilution** |
| --- | --- | --- | --- | --- | --- |
| PE-anti-mouse CD3ε | Biolegend | Flow Cytometry | 100308 | 145-2C11 | 1:10 |
| PE-anti-mouse/human CD11b | Biolegend | Flow Cytometry | 101208 | M1/70 | 1:10 |
| PE-anti-mouse IFN-γ | Biolegend | Flow Cytometry | 505808 | XMG1.2 | 1:10 |
| PerCP/Cyanine5.5 anti-mouse CD4 | Biolegend | Flow Cytometry | 100540 | RM4-5 | 1:10 |
| PE/Cyanine7 anti-mouse CD3ε | Biolegend | Flow Cytometry | 100320 | 145-2C11 | 1:10 |
| PE/Cyanine7 anti-mouse Ly-6G | Biolegend | Flow Cytometry | 127618 | 1A8 | 1:10 |
| PE/Cyanine7 anti-mouse IL-2 | Biolegend | Flow Cytometry | 503832 | JES6-5H4 | 1:10 |
| PE/Cyanine7 anti-mouse I-A/I-E（MHC-II） | Biolegend | Flow Cytometry | 107629 | M5/114.15.2 | 1:10 |
| APC anti-mouse CD8a | Biolegend | Flow Cytometry | 100712 | 53-6.7 | 1:10 |
| APC anti-mouse F4/80 | Biolegend | Flow Cytometry | 123116 | BM8 | 1:10 |
| APC anti-mouse TNF-α | Biolegend | Flow Cytometry | 506308 | MP6-XT22 | 1:10 |
| APC anti-mouse Ly-6G/Ly-6C (Gr-1) | Biolegend | Flow Cytometry | 108412 | RB6-8C5 | 1:10 |
| APC/Cyanine7 anti-mouse CD45 | Biolegend | Flow Cytometry | 103116 | 30-F11 | 1:10 |
| APC/Cyanine7 anti-mouse CD8a | Biolegend | Flow Cytometry | 100714 | 53-6.7 | 1:10 |
| Brilliant Violet 421™ anti-mouse CD3ε | Biolegend | Flow Cytometry | 100336 | 145-2C11 | 1:10 |
| Brilliant Violet 605™ anti-mouse Ly-6G | Biolegend | Flow Cytometry | 127639 | 1A8 | 1:10 |
| Purified NA/LE Hamster Anti-Mouse CD3e | BD Pharmingen | T-Cell activation | 553057 | 145-2C11 | 1:200 |
| Purified NA/LE Hamster Anti-Mouse CD28 | BD Pharmingen | T-Cell activation | 553294 | 37.51 | 1:500 |
| Rat anti Mouse F4/80 | BIO-RAD | Immunofluorescence | MCA497GA | Cl:A3-1 | 1:200 |
| Anti-rat IgG (H+L), (Alexa Fluor 555 Conjugate) | Cell Signaling Technology | Immunofluorescence | #4417 | / | 1:500 |
| METTL3 | Proteintech | western blot | 15073-1-AP | / | 1:1000 |
| PD-L1 | Proteintech | western blot | 66248-1-Ig | 2B11D11 | 1:1000 |
| YBX1 | Proteintech | western blot, ChIP, RIP | 20339-1-AP | / | 1:1000 |
| CD47 | ABclonal Technology | western blot | A11382 | ARC0584 | 1:1000 |
| GAPDH | Proteintech | western blot | 60004-1-Ig | 1E6D9 | 1:10000 |

**Table S3. Sequences of ChIRP Probes for Tug1 used in this study.**

| **Gene** | **Species** | **Serial number** | **Labelling pattern** | **Probe sequences** |
| --- | --- | --- | --- | --- |
| Tug1 | mouse | PC688 | 5'-Biotin | AGGAAAGCCTTTTAACACCACAGTCTT |
| Tug1 | mouse | PC689 | 5'-Biotin | TTCTCACGGCAAGATCAACAGTTCC |
| Tug1 | mouse | PC690 | 5'-Biotin | ATGATGGCTGAATGCTTCTTGGGTC |
| Tug1 | mouse | PC691 | 5'-Biotin | CCAATGCTGTTCTCAGAGATGCCTT |
| Tug1 | mouse | PC692 | 5'-Biotin | TGGTTGAAGCCTTGTGAGATGGTTAG |
| Tug1 | mouse | PC693 | 5'-Biotin | GAAGAGAATGCCATCCAGGTCAAACA |
| Tug1 | mouse | PC694 | 5'-Biotin | AGGAGAGGTCAAACAAACAACTTGGT |
| Tug1 | mouse | PC695 | 5'-Biotin | GGATGCTCAGAGTGTCTTCAGAATCAT |
| Tug1 | mouse | PC696 | 5'-Biotin | CACTCTGTTAAGTGAAGAGACAGGTGA |
| Tug1 | mouse | PC697 | 5'-Biotin | CTGAGTCTGATTTGCTGATTTGTGGC |
| Tug1 | mouse | PC698 | 5'-Biotin | TCCATTCACTGGTTAGATTCCCGATTC |
| Tug1 | mouse | PC699 | 5'-Biotin | TGCAGACTTGAGGTTCTAGTTATGAGT |
| Tug1 | mouse | PC700 | 5'-Biotin | TAGGGCCAGCCATACCAATAATTAAGC |
| Tug1 | mouse | PC701 | 5'-Biotin | TTCAGATGTTGAAAACAAGCAGGGTTG |
| Tug1 | mouse | PC702 | 5'-Biotin | ATTAACATTGACCCCATTGAGCCCC |
| Lac Z | E.coli | C01 | 5'-Biotin | ACCGCATATGGTGCACTCTC |
| Lac Z | E.coli | C02 | 5'-Biotin | GCGAATGGCGCCTGATGCGG |
| Lac Z | E.coli | C03 | 5'-Biotin | ATAGCGAAGAGGCCCGCACC |
| Lac Z | E.coli | C04 | 5'-Biotin | GGCGTTACCCAACTTAATCGC |
| Lac Z | E.coli | C05 | 5'-Biotin | CATGCAAGCTTGGCACTGGC |

**Table S4.** **The Primers for ChIP-qPCR are listed.**

| **Gene** | **Species** | **Forward primer** | **Reverse primer** |
| --- | --- | --- | --- |
| PD-L1-primer1 | human | TCACCAAAGTTGGGAAGTCA | GCAGGAGCATGGAGTTCTCT |
| PD-L1-primer2 | human | AGAGCACCTAGAAGTTCAGCG | ACCCTAAGGATTAAGGCTGCG |
| PD-L1-primer3 | human | AGGAAGTCACAGAATCCACGA | CGTCCCCCTTTCTGATAAAA |
| CD47-primer1 | human | TGCCGAGCTCAATGGAAAGT | AGCATCCCATTGCCACAGAA |
| CD47-primer2 | human | CGAGCGTGGGAACACAGG | CCGCTGCTGTTGACGC |
| CD47-primer3 | human | TTGCGGCCACATTTCGAACC | AACAAGTTCCATTCACGGTGGT |
